# Generation of a high quality library of bioactive filamentous actinomycetes from extreme biomes using a culture-based bioprospecting strategy

**DOI:** 10.3389/fmicb.2022.1054384

**Published:** 2023-01-19

**Authors:** Magdalena Świecimska, Patrycja Golińska, Michael Goodfellow

**Affiliations:** ^1^Department of Microbiology, Faculty of Biological and Veterinary Sciences, Nicolaus Copernicus University, Toruń, Poland; ^2^School of Natural and Environmental Sciences, Newcastle University, Newcastle upon Tyne, United Kingdom

**Keywords:** actinomycetes, systematics, antimicrobial activity, fungal phytopathogens, human pathogens, plant growth promoting bacteria, biocontrol agents

## Abstract

**Introduction:**

Filamentous actinomycetes, notably members of the genus *Streptomyces*, remain a rich source of new specialized metabolites, especially antibiotics. In addition, they are also a valuable source of anticancer and biocontrol agents, biofertilizers, enzymes, immunosuppressive drugs and other biologically active compounds. The new natural products needed for such purposes are now being sought from extreme habitats where harsh environmental conditions select for novel strains with distinctive features, notably an ability to produce specialized metabolites of biotechnological value.

**Methods:**

A culture-based bioprospecting strategy was used to isolate and screen filamentous actinomycetes from three poorly studied extreme biomes. Actinomycetes representing different colony types growing on selective media inoculated with environmental suspensions prepared from high-altitude, hyper-arid Atacama Desert soils, a saline soil from India and from a Polish pine forest soil were assigned to taxonomically predictive groups based on characteristic pigments formed on oatmeal agar. One hundred and fifteen representatives of the colour-groups were identified based on 16S rRNA gene sequences to determine whether they belonged to validly named or to putatively novel species. The antimicrobial activity of these isolates was determined using a standard plate assay. They were also tested for their capacity to produce hydrolytic enzymes and compounds known to promote plant growth while representative strains from the pine forest sites were examined to determine their ability to inhibit the growth of fungal and oomycete plant pathogens.

**Results:**

Comparative 16S rRNA gene sequencing analyses on isolates representing the colour-groups and their immediate phylogenetic neighbours showed that most belonged to either rare or novel species that belong to twelve genera. Representative isolates from the three extreme biomes showed different patterns of taxonomic diversity and characteristic bioactivity profiles. Many of the isolates produced bioactive compounds that inhibited the growth of one or more strains from a panel of nine wild strains in standard antimicrobial assays and are known to promote plant growth. Actinomycetes from the litter and mineral horizons of the pine forest, including acidotolerant and acidophilic strains belonging to the genera *Actinacidiphila,Streptacidiphilus* and *Streptomyces,* showed a remarkable ability to inhibit the growth of diverse fungal and oomycete plant pathogens.

**Discussion:**

It can be concluded that selective isolation and characterization of dereplicated filamentous actinomyctes from several extreme biomes is a practical way of generating high quality actinomycete strain libraries for agricultural, industrial and medical biotechnology.

## 1. Introduction

The phylum *Actinomycetota* (Oren and Garrity, 2021), formerly *Actinobacteria sensu* Goodfellow (2012), encompasses actinomycetes that are common in natural habitats where they have a major role in recycling organic matter (Bhatti et al., 2017) and in the transformation of environmental pollutants, such as pesticides (Alvarez et al., 2017). However, actinomycetes are known best for their unique ability to synthesise new specialised (secondary) metabolites, notably clinically significant antibiotics (Newman and Cragg, 2020; De Simeis and Serra, 2021). A new generation of antibiotics are urgently needed to control multi-drug resistant microbial pathogens which are causing a global health crisis (World Health Organization, 2021).

Around 70% of known antibiotics are produced by filamentous actinomycetes, notably by members of the genus *Streptomyces* (Sánchez-Suárez et al., 2020; Donald et al., 2022), the type genus of the family *Streptomycetaceae* Waksman and Henrici, 1943. Streptomycetes remain a rich source of new specialised metabolites, especially antibiotics (Sivalingam et al., 2019; Lacey and Rutledge, 2022) with the promise of more to come now that improved analytical procedures, such as genome mining and genetic engineering, are opening up new opportunities for drug discovery (Luo et al., 2014; Atanasov et al., 2021). However, the search for new natural products from streptomycetes using culture-dependent strategies needs to be tailored to meet developments in their ecology and systematics (Nouioui et al., 2018; Traxler and Rozen, 2022; Wang et al., 2022), as shown by the transfer of validly named acidotolerant and acidophilic *Streptomyces* species to the genera *Actinacidiphila, Streptantibioticus* and *Wenjunlia* whilst some *Streptacidiphilus* species have been moved to form the new genera *Peterkaempfera* and *Phaeacidiphilus* (Madhaiyan et al., 2022). Members of these poorly studied taxa are promising sources of new bioactive compounds (Golińska et al., 2023), as are *Kitasatospora* strains (Takahashi, 2017) which also belong to the family *Streptomycetaceae*.

In addition to therapeutic antibiotics, filamentous actinomycetes, including streptomycetes, are a valuable source of anticancer and biocontrol agents, biofertilizers, enzymes, immunosuppressive drugs and other biologically active compounds (Mukhtar et al., 2017; Sivalingam et al., 2019; Pacios-Michelena et al., 2021; Boukhatem et al., 2022). The new natural products needed for such purposes are now being sought from extreme habitats as harsh environmental conditions therein select for novel strains with distinctive genetic and molecular features, notably an ability to produce specialised metabolites of biotechnological value (Bull and Goodfellow, 2019; Sayed et al., 2020; Wilson and Brimble, 2021).

In practice, novel actinomycetes, especially streptomycetes, from extreme biomes are proving to be an especially rich source of new antibiotics, as shown by strains isolated from desert soils (Rateb et al., 2018; Djinni et al., 2019; Sun et al., 2022), deep-sea sediments (Nweze et al., 2020; Jagannathan et al., 2021) and marine organisms (Chen et al., 2021) whereas ones with growth promoting traits are being used to mitigate the effect of drought on crop plants (Chukwuneme et al., 2020). The extension of such studies to neglected extreme ecosystems can be expected to build upon these developments as biome type and geographical location are known to influence the composition of actinomycete communities (Charlop-Powers et al., 2015; Hernandez et al., 2021), not least with respect to streptomycetes (Andam et al., 2016; Arocha-Garza et al., 2017; Aallam et al., 2021). It is therefore timely to explore the taxonomic diversity and biotechnological potential of poorly studied actinomycetes from litter and mineral horizons of coniferous forests (Golińska et al., 2023) and from saline soils (Zhao et al., 2016). Improved bioinformatic tools for recognising prokaryotic species boundaries (Meier-Kolthoff et al., 2013; Sant'Anna et al., 2019) are bringing greater precision to bioprospecting campaigns, as are dereplication procedures designed to select representative isolates from extensive strain libraries for biotechnological purposes (Goodfellow et al., 2018).

Search and discovery campaigns intended to recover actinomycetes of potential biotechnological significance from extreme habitats are generally focused on the selective isolation, preliminary characterisation and antimicrobial screening of small numbers of isolates from individual biomes (Liu et al., 2016; Priyadarshini et al., 2016; Meklat et al., 2020). This partly reflects a tension between the need to screen representative isolates for new metabolites and the requirement to classify them using taxonomic methods that are difficult to apply to more than a few strains. This problem is being addressed by using dereplication procedures, such as MALDI-TOF mass spectrometry (Schumann and Maier, 2014), molecular fingerprinting (Carro et al., 2018) and genus specific primers (Castro et al., 2019), to distribute large numbers of isolates to taxonomically meaningful groups, representatives of which can be selected for further study. A practical and inexpensive way of dereplicating streptomycetes was introduced by Williams et al. (1969) who assigned large numbers of soil streptomycetes to colour-groups based on aerial spore mass, substrate mycelial and diffusible pigment colours formed on oatmeal agar and melanin pigments produced on peptone-yeast extract – iron agar. Subsequently, the number of colour-groups were used as an index of streptomycete diversity in diverse natural habitats (Atalan et al., 2000; Sembiring et al., 2000; Antony-Babu et al., 2010) following the discovery that representatives of such groups belonged to validly named or novel (previously unknown) *Streptomyces* species or species-groups based upon genotypic and phenotypic criteria (Manfio et al., 2003; Goodfellow et al., 2007), as exemplified by members of what became known as the *Streptomyces violaceusniger* clade (Sembiring et al., 2000; Goodfellow et al., 2007. Kusuma et al., 2021). Representatives of this taxon show a similar pattern of HPLC detected antibiotics (Ward and Goodfellow, 2014). Recently, principal component analyses of members of colour-groups composed of desert filamentous actinomycetes were shown to be positively correlated with corresponding levels of bioactivity recorded from antimicrobial assays (Goodfellow et al., 2018). The colour-group strategy has been extended to include representatives of other genera containing filamentous actinomycetes (Idris, 2016; Kusuma, 2020).

In the present study, filamentous actinomycetes isolated from hyper-arid, high altitude Atacama Desert soils, a saline soil and from two pine forest locations were assigned to colour-groups in order to gain an insight into the generic diversity at each of the sampling sites. 16S rRNA gene sequencing analyses were carried out on representatives of the colour-groups to determine whether they belonged to validly named species or to putatively new species and the resultant phylogenetic data used to establish the taxonomic identity of isolates assigned to the colour-groups. The antimicrobial activity of colour-group representatives was determined using a standard plate assay, as was their ability to produce hydrolytic enzymes and compounds known to promote plant growth. In addition, representative strains from the pine forest sites were examined to determine their ability to inhibit the growth of fungal and oomycete plant pathogens. The overall aim of the study was to compare and contrast the taxonomic and functional activities of representative isolates from the four sampling sites and to generate a high quality strain library for agricultural, medical and industrial biotechnology.

## 2. Materials and methods

### 2.1. Sampling sites, selective isolation and maintenance of isolates

Filamentous actinomycetes were isolated from environmental samples taken from high altitude, hyper-arid, Atacama Desert soils on Cerro Chajnantor, Chile (Idris et al., 2017a,b; Bull et al., 2018), from litter and mineral layers of an acidic forest soil under *Pinus sylvestris*, near Toruń, Poland (Golińska et al., 2016; Świecimska et al., 2021a,b) and from a saline soil adjacent to Lake Lonar, India (Świecimska et al., 2020); these references include details on the sampling sites, their location and physico-chemical properties and on isolation procedures. Tenfold dilutions of the Atacama Desert soil samples were used to inoculate starch-casein agar (SCA; Küster and Williams, 1964), humic acid-vitamin agar (HA; Hayakawa and Nonomura, 1987), Gauze’s no. 1 agar (G; Zakharova et al., 2003) and R2A agar (Becto-Dickinson, United State) plates which were incubated at 28°C for 21 days. The pH of the Atacama soil samples ranged from 6.6 to 7.6, the organic matter content from 1.7% to 3.7%, the moisture content was zero %.

In total, the 226 representative actinomycetes consisted of 59 isolates from the hyper-arid Atacama Desert soils and 16 from the saline soil, including 3 classified as *Streptomyces alkaliterrae* by Świecimska et al. (2020). Similarly 151 isolates from the litter and mineral layers of the pine forest soils comprised 65 and 86 strains from the northern and southern slopes of the inland dune system supporting pine, including pairs of strains classified as *Catenulispora pinisilvae* (Świecimska et al., 2021a) and *Catenulispora pinistramenti* (Świecimska et al., 2021b). The isolates from the desert soils included 6 classified as either *Modestobacter altitudinis* (Golińska et al., 2020a) or *Modestobacter excelsi* (Golińska et al., 2020b) and the type strain of *Micromonosporra acroterricola* (Carro et al., 2019). Isolates were taken from the selective isolation plates based on characteristic colonial properties. Isolates from the Atacama Desert soils were maintained on slopes of modified Bennett’s agar (Jones, 1949), ones from the saline soil on halophilic nutrient agar (Atlas, 2010) and those from the pine forest sites on SCA slopes, at room temperature and as suspensions of spores and mycelial fragments or as rods and cocci in 20%, v/v glycerol at −80°C.

### 2.2. Assignment of isolates to colour-groups

The representative isolates were grown for 4 weeks at 28°C on oatmeal agar [International *Streptomyces* Project (ISP) medium 3] (Shirling and Gottlieb, 1966) at pH 5.5, 7.5 and 8.0 in the case of isolates from the pine forest, saline and Atacama Desert sampling sites; the ISP3 medium was adjusted to pH 5.5 using 1 M HCl. The isolates were then assigned to four sets of colour-groups, representing the desert, saline and the two pine forests sites based on aerial spore mass, substrate mycelial and diffusible pigment colours using NBS/IBCC Colour Charts (Kelly, 1958). All of the isolates were grown on peptone-yeast extract-iron agar (ISP6, Shirling and Gottlieb, 1966) under the conditions described above and then examined to determine whether they produced melanin pigments.

### 2.3. Phylogenetic analyses

Most of the 99 isolates representing the colour-groups composed of 71 strains from the pine forest sites and 28 from the Atacama Desert soils were grown in ISP2 broth (Shirling and Gottlieb, 1966) at pH 5.5 and 7.5, respectively, the exceptions, desert isolates 1G4^T^, 1G6^T^, 1G14 and 1G50 to 1G52 were cultivated at pH 7.5 in modified Bennett’s broth (Jones, 1949). The 16 isolates representing the colour-groups containing strains from the saline soil were grown at pH 8.0 in halophilic nutrient broth (Atlas, 2010) supplemented with 3% NaCl. All of the isolates were grown in shake flasks (150 rpm) at 28°C for 14–21 days, harvested by centrifugation, washed three times with sterile distilled water and stored at room temperature.

Genomic DNA was extracted from the biomass samples using a GenElute™ Bacterial Genomic Kit (Sigma-Aldrich, Germany), according to the manufacturer’s instructions. Amplification of 16S rRNA genes was performed using standard forward (27f: AGAGTTTGATCCTGGCTCAG) and reverse (1525r, AAGGAGGTGATCCAGCC) primers (Lane, 1991) under the following conditions (25 μL volume): 2x MyFi Mix (Bioline, United Kingdom) which contained DNA polymerase, dNTPs, MgCl_2_ (at optimised concentrations), and 1 μL of each primer (20 μM), 1 μL of 200 ng DNA and sterile distilled water; the negative control was sterile distilled water and the positive one DNA isolated from *Actinospica durhamensis* DSM 46820^T^. The PCR reactions were carried out as follows: initial denaturation at 95°C for a minute, 30 cycles of 95°C for a minute, 55°C for a minute and 72°C for a minute, and finally 72°C for 5 min. The PCR products were purified using a purification kit (Qiagen, Germany), according to the instructions of the manufacturer. The concentration and quality of each of the purified PCR products were checked using a Nanodrop spectrophotometer (NanoDrop 2000, Thermo Fisher Scientific, United State) and by gel electrophoresis using a 1 kb DNA ladder (Kapa Biosystems, United State). The resultant preparations were sequenced on an ABI 3730xl Genetic Instrument (Applied Biosystems, Thermo Fisher Scientific United State) at the Institute of Biochemistry and Biophysics of the Polish Academy of Sciences in Warsaw using the same pair of primers, as given above. Almost complete 16S rRNA gene sequences of the isolates were identified using a combination of two sequence similarity search engines (blast and megablast; Altschul et al., 1997), followed by rigorous pairwise global sequence alignment (Myers and Miller, 1988), as previously described (Chun et al., 2007), as implemented through the EzBioCloud web server^1^ (Yoon et al., 2017). Isolates showing 16S rRNA gene sequences equal to or <99.0% (maximum probability of error 1.0%) with their immediate phylogenetic neighbours were considered to belong to putatively new species (Meier-Kolthoff et al., 2013).

### 2.4. Antimicrobial activity

The 115 isolates representing the colour-groups composed of strains from the four sampling sites were examined for their ability to inhibit the growth of *Bacillus subtilis* PCM 2021, *Escherichia coli* PCM 2057, *Klebsiella pneumoniae* ATCC 700603, *Micrococcus luteus* ATCC 10240, *Proteus mirabilis* CM NCU (isolate from Collegium Medicum Nicolaus Copernicus University), *Pseudomonas aeruginosa* ATCC 10145, *Salmonella infantis* SES (isolate from the Sanitary-Epidemiological Station in Toruń, Poland), *Staphylococcus aureus* PCM 2054 and *Candida albicans* ATCC 10231 using a standard agar plug method (Fiedler, 2004), as described by Świecimska et al. (2022). Briefly, the isolates from the Atacama Desert and pine forest samples were grown on ISP2 and ISP3 agar (Shirling and Gottlieb, 1966) at pH 7.5 and 5.5, respectively, and those from the saline soil adjacent to Lake Lonar on HA agar (Atlas, 2010) supplemented with 3% NaCl and on ISP2 agar (Shirling and Gottlieb, 1966), pH 8.0, following incubation for 3 weeks at 28°C. Plugs (ø = 5 mm) of each of the isolates were cut aseptically using a sterile cork borer, and placed in square Petri dishes (120 × 120 mm). Overnight cultures of the reference bacteria and the yeast grown at 37°C in tryptic soy broth (TSB, Becton Dickinson, United State) and Sabouraud dextrose broth (SDB, Becton Dickinson, United State), respectively, were used to prepare inocula in Luria Bertani broth (LB, Becton Dickinson, United State) at an optical density (OD) of 0.6. These inocula were used to seed LB broths prior to diluting them with the same volume of nutrient agar (NA, Becton Dickinson, United State). In all cases, the final concentration of the reference microorganisms was 1.5–2 × 10^6^ CFU per mL. Each of these media were poured into the plates containing the agar plugs and the resultant preparations incubated for 24 h at 37°C when inhibition zones around the agar plugs were measured in mm. All of the tests were carried out in triplicate. The data acquired from the three experiments were presented as mean values ± standard deviations (SD).

### 2.5. Screening against fungal and oomycete plant pathogens

These experiments were restricted to 71 isolates representing colour-groups composed of strains from the acidic forest samples; the isolates from the desert and saline soils were not considered as they did not grow optimally, if at all, at pH 5.5–6.0, the required range for the antifungal assays. The pine forest isolates were tested for their ability to inhibit the growth of fungal and oomycete plant pathogens using the co-culture method, as described by Świecimska et al. (2021a, 2022). To this end, the isolates were streaked on the right side of Petri plates of potato dextrose agar (PDA, Becton Dickinson, United State) and incubated for 2 weeks at 28°C. These preparations were inoculated at the opposite side of the Petri plates with the agar plugs (ø = 8 mm). The pathogenic fungi and oomycetes, namely *Phytophthora cactorum, Phytophthora cryptogea*, *Phytophthora megasperma* and *Phytophthora plurivora*, were grown on PDA for 7–21 days at 28°C. The co-cultures were incubated for 7 days in the case of those involving *Alternaria alternata* IOR 1783 (isolated from kohlrabi), *Fusarium culmorum* IOR 2333 (isolated from a pine root), *Fusarium culmorum* D (isolated from wheat), *Phytophthora plurivora* IOR 2303 (isolated from oak rhizosphere), *Rhizoctonia solani* 13 (isolated from a pine root) and *Sclerotina sclerotiorum* IOR 2242 (isolated from broccoli); for 14 days with respect to those involving *Botritis cinerea* IOR 1873 (isolated from tomato), *Colletotrichum acutatum* IOR 2153 (isolated from blueberry), *Fusarium oxysporum* IOR 342 (isolated from pine), *Fusarium poae* A and *Fusarium tricinctum* A (isolated from wheat) and *Phytophthora cactorum* IOR 1925 (isolated from strawberry), and for 21 days in corresponding preparations involving *Fusarium graminearum* A and *Fusarium oxysporum* D (isolated from wheat), *Fusarium solani* IOR 825 (isolated from parsley), *Phytophthora cryptogea* IOR 2080 (isolated from Lawson cypress), *Phytophthora megasperma* IOR 404 (isolated from raspberry), and *Phoma lingam* IOR 2284 (isolated from rape). All of the tests were carried out in triplicate at 28°C. Activity against *Chalara fraxinea* (isolate from ash) was tested using the same procedure, but on malt extract agar (MEA; 20 g L^−1^, malt extract, 15 g L^−1^ agar; Kowalski, 2006) with incubation for 8 weeks at room temperature. The negative controls were cultures of the fungal and oomycete pathogens grown under the same incubation conditions. Inhibition (I) of pathogen growth was calculated using the formula: I (%) = (C-T/C) × 100, where C is the diameter of pathogen growth in the control sample and T the diameter of pathogen growth in each of the co-culture samples. Data obtained from *in vitro* experiments were reported as mean values ± standard deviations (SD).

### 2.6. Promotion of plant growth

The representative isolates were tested, in triplicate, for their ability to produce ammonia, indol-3-acetic acid (IAA) and hydrogen cyanide (HCN) using ISP2 medium (broth and agar, respectively; Shirling and Gottlieb, 1966), at pH 5.5 and 7.5, as the basal media for the isolates representing the desert and pine forest colour-groups, respectively, whereas halophilic nutrient broth (Atlas, 2010), pH 8.0, supplemented with 3% sodium chloride was the basal medium used for the representatives of the colour-groups containing strains from the saline soil.

All of the isolates were examined for their ability to produce ammonia using the modified method described by Cappucino and Sherman (1992) and the basal media supplemented with L-tryptophan (5 mg mL^−1^); flasks containing the inoculated media were shaken (150 rpm) for 14–21 days at 28°C then centrifuged (10,000 rpm for 10 min). One mL of each of the resultant supernatants was mixed with 0.5 mL of Nessler’s reagent; the development of a yellow to brown colour indicated that ammonia had been formed. Similarly, the production of IAA was detected using the modified method described by Brick et al. (1991); the inoculated basal broth cultures supplemented with L-tryptophan (5 mg mL^−1^) were shaken (150 rpm) for 14–21 days at 28°C, centrifuged at 10,000 rpm for 10 min and aliquots of the supernatants (50 μL) mixed with 100 μL Salkowski reagent (49 mL of 35% perchloric acid and 1 mL of 0.5 M FeCl_3_ solution) and the preparations incubated in the dark for 30 min. The development of a pink colour in the resultant preparations indicated the presence of IAA; the negative control consisted of the corresponding growth media mixed with Salkowski reagent.

The isolates were also examined for their ability to solubilise phosphate using a medium containing 10 g glucose, 0.5 g NH_4_SO_4_, 0.8 g K_2_HPO_4_, 0.2 g KH_2_PO_4_, 0.2 g NaCl, 0.1 g KCl, 0.3 g MgSO_4_ · 7H_2_O, 2 g yeast extract, 2.5 g Ca_3_(PO_4_)_2_, 0.5 g arabic gum and 20 g agar; the arabic gum was used to keep the Ca_3_(PO_4_)_2_ in suspension. The width of colonies and hydrolysis zones were measured in millimetres after 14 days of incubation at 28°C and activity indices calculated as follows: Wact = Sh^2^ (Sc × t) where Sh indicates the diameter of the hydrolysis zone, Sc the colony diameter and t the time of incubation (Hrynkiewicz et al., 2010).

The production of HCN was determined after Lock (1948). The isolates were grown on slopes of the basal agar media supplemented with glycine (4.4 g L^−1^) and the inoculated tubes incubated for 7 days at 28°C when Whatman filter paper (Merck) strips soaked with 2% sodium carbonate in 0.5% picric acid solution were inserted into the neck of the tubes; the latter were sealed with parafilm and incubated in the dark for 14 days at 28°C. A colour change on the paper strips from yellow to brown indicated that HCN had been produced. Similarly, the ability of the isolates to synthesis siderophores was established using the method of Alexander and Zuberer (1991); the isolates were inoculated onto Chrome Azurol S (CAS) medium and incubated at 28°C for 14 days. A colour change in the media from blue to orange under and around the colonies indicated the presence of siderophores; the resultant activity indices were estimated, as described above. Both of these tests were conducted three times.

### 2.7. Synthesis of hydrolytic enzymes

The isolates representing the various colour-groups were examined to establish whether they synthesised a range of hydrolytic enzymes of ecological and potential industrial importance. Strains isolated from the desert, pine forest and saline environmental samples were grown on dedicated media, given below, at pH 7.5, 6.5, and 7.5, respectively. In all cases the isolates were inoculated at the centre of the agar plates; the latter were incubated for 14 days at 28°C when the width of hydrolysis zones around colonies were measured and coefficients of activity determined, as described above. The ability of the isolates to produce cellulases and chitinases were determined according to Berg and Pettersson (1977) and Lingappa and Lockwood (1962), respectively. The production of cellulases was established by flooding incubated plates with a 0.1% solution of Congo red for 15 min and then with 1 M NaCl for 15 min; hydrolysis zones appeared as orange haloes against a red background. Lipolytic and pectinolytic activities were determined using procedures described by Gibson and Gordon (1974) and Strzelczyk and Szpotański (1989), respectively. Hydrolysis of pectin was detected by flooding incubated plates with 10% Cetrimide solution for 15 min; zones of clearing around colonies indicated positive results. Proteolytic activity was tested on a medium containing 5 g powdered skimmed milk, 0.1 g (NH_4_)_2_SO_4_, 0.1 g FeSO_4_ · 7H_2_O, 0.1 g yeast extract and 15 g agar. Clear zones around colonies were recorded as activity indices. Finally, urease activity was detected after Tidwell et al. (1955); changes in the colour of the medium from yellow to orange-pink were recorded as positive results. All tests for synthesis of hydrolytic enzymes were performed in triplicate.

## 3. Results

### 3.1. Colour-group assignment and dereplication of isolates

Most of the representative strains from the selective isolation plates formed extensively branched substrate mycelia bearing either aerial hyphae or distinct masses of coloured spores characteristic of the genus *Streptomyces*. The 59 isolates representing the Atacama Desert colour-groups were assigned to one single-and 12 multi-membered colour-groups with between 3 and 7 isolates, as shown in Supplementary Table S1A. None of the isolates formed diffusible pigments or melanin pigments and those belonging to colour-groups 2 and 4 did not produce aerial hyphae. Colour-group 1, one of the two largest taxa, consisted of isolates that exhibited a medium grey aerial spore mass and a moderate yellow green substrate mycelium. The 16 isolates from the saline soil fell into 4 single-and 4 multi-membered colour-groups with between 2 and 5 strains, all but one of which included isolates which produced characteristic diffusible pigments (Supplementary Table S1B); none of the isolates formed melanin pigments. The largest taxon, colour-group 1, included isolates which formed a distinctive moderate yellow substrate mycelium and a characteristic pale purple diffusible pigment, but not aerial hyphae. The most extensive taxonomic variation was found amongst the 65 strains isolated from the northern slope of the inland pine dune. These isolates were assigned to 7 single-and 14 multi-membered colour-groups with between 2 and 9 strains all of which formed aerial hyphae, apart from isolates belonging to the first colour-group; many of these isolates produced characteristic diffusible pigments, as shown Supplementary Table S1C. Similarly, the 86 isolates from the southern inland pine dune fell into 6 single-and 12 multi-membered colour-groups with between 2 and 24 strains, all of which formed aerial hyphae (Supplementary Table S1D). Colour-group 1, the largest taxon, consisted of 24 isolates with distinctive features, a moderate yellow green aerial spore mass, brilliant greenish yellow substrate mycelia and brilliant yellow green diffusible pigments. Only two strains produced melanin pigments on PYEA, namely isolates NL30 and SL26 from the northern and southern sampling sites in the pine forest.

### 3.2. Taxonomic diversity of representative isolates

The 115 isolates representing the four sets of colour-groups are shown in Table 1 together with their closest phylogenetic neighbours based on 16S rRNA gene sequence similarities. Twenty-eight of the isolates (24%) shared sequence similarities with their nearest neighbours at or below the 99.0% species threshold, as exemplified by isolates NL23, SA10, 2SCA1, SF9, IF12, SL55, NL15 and NH28; these isolates can be considered to be putatively novel species of *Actinacidiphila*, *Actinospica, Kribbella, Nocardia, Nocardiopsis, Pilimelia, Streptacidiphilus*, and *Streptomyces*, respectively. In contrast, representatives of several genera showed identical or almost identical sequences with the type strains of validly named species, as illustrated by isolates NF3 and NF23 which shared 100% and 99.86% gene sequence similarities with the type strains of *C. pinisilvae* and *C. pinistramenti*, respectively. This was also the case with isolates 1G51, 1G52, and 1G14 (100% sequence similarities) and 1G50 (99.86% sequence similarity) with the corresponding type strains of *M. altitudinis* and *M. excelsi*. Similarly, identical or almost identical sequence similarities were found between isolate 5R2A3 and *M. acroterricola* 5R2A7^T^, isolate OF4 and *Nocardiopsis metallicus* KBS6^T^ and isolate NF10 and *Streptacidiphilus torunensis* NF37^T^. The most numerous strains showing identical 16S rRNA gene sequences were between isolates and the type strains of *Streptomyces* species, as exemplified by isolates OF2, OF3, OF7, and OF8 and *S. alkaliterrae* OF1^T^, isolate NH5 and *S. atratus* NRRL B-16927^T^, and isolates SF4, SF8, SH11, and SL3 and *S. celluloflavus* NRRL B-2493^T^. Isolates assigned to the genus *Streptomyces* predominated within each of the four sets of colour-groups, as shown in Table 1.

**Table 1:** Nearest neighbours of isolates based on 16S rRNA gene sequence similarities using the EzBioCloud server (Yoon et al., 2017).

**Isolates**	**Nearest neighbours**	**Codes of type strains**	**Sequence similarity**	**Nucleotide differences/total number of nucleotides**	**Length of 16S rRNA genes**
(A) Representative isolates from the hyper–arid Atacama Desert soils					
2G7	*Kribbella flavida*	DSM 17836^T^	99.43	8/1,411	1,416
1HA1	*Kribbella italica*	BC637^T^	99.36	9/1,411	1,416
**2SCA1**	***Kribbella turkmenica***	**16K104** ^T^	**98.94**	**15/1,413**	**1,417**
5R2A3	*Micromonospora acroterricola*	5R2A7^T^	99.72	4/1,407	1,411
5R2A7^T^*	*M. acroterricola*	5R2A7^T^	100	0/1,413	1,413
1G4^T^*	*Modestobacter altitudinis*	1G4^T^	100	0/1,526	1,526
1G51*	*M. altitudinis*	1G4^T^	100	0/1,397	1,398
1G52*	*M. altitudinis*	1G4^T^	100	0/1,391	1,394
1G6^T^*	*Modestobacter excelsi*	1G6^T^	100	0/1,526	1,526
1G14*	*M. excelsi*	1G6^T^	100	0/1,408	1,416
1G50*	*M. excelsi*	1G6^T^	99.86	2/1,410	1,417
1HA3	*Pseudonocardia khuvsgulensis*	MN08-A0297^T^	99.36	9/1,399	1,399
2R2A4	*Pseudonocardia rhizophila*	YIM 67013^T^	99.72	4/1,411	1,416
4R2A1	*P. rhizophila*	YIM 67013^T^	99.64	5/1,403	1,403
**3G5**	***Pseudonocardia xinjiangensis***	**AS 4.1538** ^**T**^	**97.81**	**29/1,325**	**1,408**
5SCA4	*Streptomyces albogriseolus*	NRRL B-1305^T^	99.43	8/1,412	1,412
1R2A7	*Streptomyces aquilus*	GGCR-6^T^	99.28	10/1,393	1,405
3G6	*Streptomyces bungoensis*	DSM 41781^T^	99.51	7/1,419	1,423
**4SCA5**	***Streptomyces camponoticapitis***	**2H-TWYE14** ^**T**^	**97.58**	**34/1,407**	**1,412**
1G2	*Streptomyces flaveolus*	NBRC 3715^T^	99.72	4/1,419	1,424
1R2A1	*S. flaveolus*	NBRC 3715^T^	99.72	4/1,411	1,411
3HA10	*Streptomyces galbus*	DSM 40089^T^	99.37	9/1,419	1,424
5SCA5	*Streptomyces marokkonensis*	Ap1^T^	99.50	7/1,412	1,412
5SCA7	*S. marokkonensis*	Ap1^T^	99.50	7/1,412	1,412
5HA2	*Streptomyces mutabilis*	NBRC 12800^T^	99.79	3/1,422	1,426
**2G9**	***Streptomyces paradoxus***	**NBRC 14887** ^T^	**98.79**	**17/1,405**	**1,411**
1SCA19	*Streptomyces purpurascens*	NBRC 13077^T^	99.15	12/1,411	1,415
1SCA21	*Streptomyces tendae*	ATCC 19812^T^	99.15	12/1,411	1,411
(B) Representative isolates from saline soil adjacent to Lake Lonar					
**IT2**	***Nocardiopsis flavescens***	**CGMCC 4.5723** ^**T**^	**97.45**	**36/1,412**	**1,415**
**IF12**	***Nocardiopsis halotolerans***	**DSM 44410** ^**T**^	**98.72**	**18/1,410**	**1,411**
OF4	*Nocardiopsis metallicus*	KBS6^T^	100	0/1,419	1,417
OF6	*N. metallicus*	KBS6^T^	99.86	2/1,417	1,417
OT1	*Nocardiopsis valliformis*	DSM 45023^T^	99.50	7/1,413	1,421
IF11*	*Streptomyces alkaliphilus*	DSM 42118^T^	99.42	8/1,387	1,389
IF15	*S. alkaliphilus*	DSM 42118^T^	99.36	9/1,414	1,414
IF17*	*S. alkaliphilus*	DSM 42118^T^	99.52	7/1,451	1,535
IF19*	*S. alkaliphilus*	DSM 42118^T^	99.50	7/1,402	1,402
OF1^T^*	*Streptomyces alkaliterrae*	OF1^T^	100	0/1,534	1,534
OF2	*S. alkaliterrae*	OF1^T^	100	0/1,406	1,406
OF3*	*S. alkaliterrae*	OF1^T^	100	0/1,450	1,534
OF5	*S. alkaliterrae*	OF1^T^	99.79	3/1,413	1,413
OF7	*S. alkaliterrae*	OF1^T^	100	0/1,401	1,401
OF8*	*S. alkaliterrae*	OF1^T^	100	0/1,450	1,534
**IF7**	***Streptomyces cahuitamycinicus***	**13K301** ^**T**^	**97.24**	**39/1,411**	**1,411**
(C) Representative isolates from the litter and mineral horizons of the northern slope of inland dune of the pine forest					
**NH28a**	***Actinacidiphila alni***	**D65** ^**T**^	**98.50**	**21/1,403**	**1,403**
**NL16**	***Actinacidiphila bryophytorum***	**NEAU-HZ10** ^**T**^	**98.48**	**21/1,386**	**1,388**
**NL23**	***Actinacidiphila paucisporea***	**CGMCC 4.2025** ^**T**^	**98.62**	**19/1,377**	**1,385**
**NF27**	***Actinacidiphila yanglinensis***	**1307** ^**T**^	**98.14**	**26/1,395**	**1,406**
NH21	*A. yanglinensis*	1307^T^	99.07	13/1,401	1,406
NH27	*A. yanglinensis*	1307^T^	99.22	11/1,408	1,415
NH11^T^*	*Catenulispora pinisilvae*	NH11^T^	100	0/1,441	1,525
NF3*	*C. pinisilvae*	NH11^T^	100	0/1,525	1,525
NL13	*C. pinisilvae*	NH11^T^	99.79	3/1,401	1,402
NF23*	*Catenulispora pinistramenti*	NL8^T^	99.86	2/1,441	1,519
NL8 ^T^*	*C. pinistramenti*	NL8^T^	100	0/1,398	1,398
NF24	*Kitasatospora herbaricolor*	NBRC 12876^T^	99.71	4/1,403	1,403
NF39	*K. herbaricolor*	NBRC 12876^T^	99.64	5/1,402	1,402
NH9	*K. herbaricolor*	NBRC 12876^T^	99.43	8/1,404	1,408
NL28	*Pilimelia columellifera subsp. pallida*	MB-SK 8^T^	99.64	5/1,408	1,420
**NA4**	***Streptacidiphilus albus***	**NBRC 100918** ^**T**^	**97.73**	**31/1,364**	**1,371**
**NL15**	***Streptacidiphilus carbonis***	**DSM 41754** ^**T**^	**98.44**	**22/1,407**	**1,422**
NA13	*Streptacidiphilus durhamensis*	FSCA67^T^	99.64	5/1,385	1,417
NA19a	*S. durhamensis*	FSCA67^T^	99.86	2/1,382	1,383
**NA21**	***Streptacidiphilus hamsterleyensis***	**HSCA 14** ^**T**^	**92.01**	**109/1,365**	**1,406**
NF22	*S. hamsterleyensis*	HSCA 14^T^	99.56	6/1,361	1,403
NH22	*Streptacidiphilus neutrinimicus*	DSM 41755^T^	99.78	3/1,377	1,383
NF10	*Streptacidiphilus torunensis*	NF37^T^	100.00	0/1,381	1,412
NF20	*S. torunensis*	NF37^T^	99.86	2/1,380	1,401
NH14	*S. torunensis*	NF37^T^	100.00	0/1,381	1,422
NH5	*Streptomyces atratus*	NRRL B-16927^T^	100.00	0/1,425	1,435
NH16	*S. atratus*	NRRL B-16927^T^	99.79	3/1,419	1,424
NA10a	*Streptomyces celluloflavus*	NRRL B-2493^T^	100.00	0/1,408	1,408
NH7	*S. celluloflavus*	NRRL B-2493^T^	99.86	2/1,398	1,398
NL3	*S. celluloflavus*	NRRL B-2493^T^	100.00	0/1,418	1,425
**NH17**	***Streptomyces luteireticuli***	**NBRC 13422** ^**T**^	**92.32**	**107/1,393**	**1,431**
**NH28**	***Streptomyces paludis***	**GSSD-12** ^**T**^	**98.13**	**26/1,390**	**1,390**
NA24	*Streptomyces xanthochromogenes*	NRRL B-5410 ^T^	100.00	0/1,404	1,404
NL21	*S. xanthochromogenes*	NRRL B-5410 ^T^	99.93	1/1,412	1,412
NH15	*Streptomyces yanii*	NBRC 14669^T^	99.20	11/1,377	1,397
NL35	*S. yanii*	NBRC 14669^T^	99.12	12/1,366	1,409
(D) Representative isolates from the litter and mineral horizons of the southern slope of inland dune of the pine forest					
**SL7**	***Actinacidiphila bryophytorum***	**NEAU-HZ10** ^**T**^	**98.33**	**23/1,381**	**1,390**
**SL52**	***A. bryophytorum***	**NEAU-HZ10** ^**T**^	**98.61**	**19/1,363**	**1,368**
**SL13**	***Actinacidiphila rubida***	**13C15** ^**T**^	**97.49**	**35/1,394**	**1,408**
SA23	*Actinacidiphila yanglinensis*	1307^T^	99.36	9/1,416	1,425
SF17	*A. yanglinensis*	1307^T^	99.50	7/1,400	1,400
**SL5**	***A. yanglinensis***	**1307** ^**T**^	**98.70**	**18/1,380**	**1,387**
**SL10**	***A. yanglinensis***	**1307** ^**T**^	**98.84**	**16/1,375**	**1,381**
**SL22**	***A. yanglinensis***	**1307** ^**T**^	**98.78**	**17/1,392**	**1,399**
**SA10**	***Actinospica acidiphila***	**GE134766** ^**T**^	**98.07**	**27/1,400**	**1,406**
**SF9**	***Nocardia nova***	**NBRC 15556** ^**T**^	**98.85**	**16/1,397**	**1,399**
**SF13**	***Nocardia vinacea***	**NBRC 16497** ^**T**^	**98.22**	**25/1,407**	**1,413**
**SH20**	***N. vinacea***	**NBRC 16497** ^**T**^	**98.26**	**24/1,378**	**1,384**
SA4	*Pilimelia columellifera* subsp. *pallida*	MB-SK 8^T^	99.71	4/1,398	1,403
SF15	*P. columellifera* subsp*. pallida*	MB-SK 8^T^	99.71	4/1,398	1,403
SF23	*P. columellifera* subsp*. pallida*	MB-SK 8^T^	99.71	4/1,398	1,401
SH24	*P. columellifera* subsp*. pallida*	MB-SK 8^T^	99.71	4/1,396	1,412
SL4	*P. columellifera* subsp*. pallida*	MB-SK 8^T^	99.72	4/1,408	1,410
SL16	*P. columellifera* subsp*. pallida*	MB-SK 8^T^	99.71	4/1,398	1,414
SL19	*P. columellifera* subsp*. pallida*	MB-SK 8^T^	99.71	4/1,398	1,414
SL24	*P. columellifera* subsp*. pallida*	MB-SK 8^T^	99.71	4/1,398	1,414
**SL55**	***P. columellifera* *subsp. pallida***	**MB-SK 8** ^**T**^	**99.00**	**14/1,406**	**1,417**
SF10	*Streptacidiphilus torunensis*	NF37^T^	100.00	0/1,381	1,418
SA20	*Streptomyces atratus*	NRRL B-16927^T^	99.79	3/1,419	1,425
SA7	*Streptomyces celluloflavus*	NRRL B-2493^T^	99.79	3/1,422	1,430
SF4	*S. celluloflavus*	NRRL B-2493^T^	100.00	0/1,404	1,404
SF8	*S. celluloflavus*	NRRL B-2493^T^	100.00	0/1,404	1,404
SH11	*S. celluloflavus*	NRRL B-2493^T^	100.00	0/1,419	1,425
SH15	*S. celluloflavus*	NRRL B-2493^T^	99.79	3/1,401	1,402
SL3	*S. celluloflavus*	NRRL B-2493^T^	100.00	0/1,423	1,430
SH56	*Streptomyces cocklensis*	BK168^T^	99.15	12/1,404	1,409
**SL54**	***Streptomyces ferralitis***	**SFOp68** ^**T**^	**97.65**	**33/1,406**	**1,411**
SH57	*Streptomyces halstedii*	NBRC 12783^T^	99.79	3/1,419	1,427
SF28^T^*	*Streptomyces pinistramenti*	SF28^T^	100	0/1,404	1,404
SA8	*Streptomyces sanglieri*	NBRC 100784^T^	100.00	0/1,401	1,401
SA16	*S. sanglieri*	NBRC 100784^T^	99.79	3/1,419	1,426

Codes and names in bold indicate isolates with 16S rRNA sequence similarity at or below the 99.0% threshold for delineating actinomycete species.

*Isolates recently classified as new species: 1G4^T^, 1G51, and 1G52 (Golińska et al., 2020b); 1G6^T^, 1G14, and 1G50 (Golińska et al., 2020a); IF11, IF17, IF19, OF1^T^, OF3, and OF8 (Świecimska et al., 2020); NF3 and NH11^T^ (Świecimska et al., 2021a); NF23 and NL8^T^ (Świecimska et al., 2021b); SF28^T^ (Świecimska et al., 2022) and 5R2A7^T^ (Carro et al., 2019).

Codes for the isolates from the Atacama Desert soils: numbers 1–5 at the beginning of the code indicate the sample sites, G, HA, R2A, SCA the isolation medium and then the isolate number. Codes: NA, NL, NF and NH/SA, SL, SF and SH refer to isolates from the mineral, litter, fermentation and humus layers of the northern and southern slopes of the inland dune of the pine forest. ^T^, type strain.

The 28 isolates from the hyper-arid desert soils included in the 16S rRNA gene sequencing analyses were assigned to the genera *Kribbella* (3 strains), *Micromonospora* (2 strains), *Modestobacter* (6 strains), *Pseudonocardia* (4 strains) and *Streptomyces* (13 strains); the codes for the Atacama Desert isolates are given in the footnote to Table 1A. Similarly, the 16 isolates from the saline soil were found to belong to the genera *Nocardiopsis* (5 isolates) and *Streptomyces* (11 isolates). Isolates from the northern slope of the inland pine dune were assigned to the genera *Actinacidiphila* (6 strains), *Catenulispora* (5 strains), *Kitasatospora* (3 strains), *Pilimelia* (1 strain), *Streptacidiphilus* (10 strains) and *Streptomyces* (11 strains), and those from the corresponding southern slope to the genera *Actinacidiphila* (8 strains), *Actinospica* (1 strain), *Nocardia* (3 strains), *Pilimelia* (9 strains), *Streptacidiphilus* (1 strain) and *Streptomyces* (13 strains). The codes for the pine forest isolates in Tables 1C,D show that they were isolated from litter, fermentation, humus and mineral horizons.

### 3.3. Identification of isolates assigned to colour-groups

In general, isolates belonging to colour-groups can be assigned to genera given the distribution of reference isolates included in the 16S rRNA gene sequencing analyses (Supplementary Table S2). In the case of the Atacama Desert isolates, for instance, isolates comprising colour-groups 2 and 5 can be considered to belong to the genera *Micromonospora* and *Kribbella*, respectively, as they include reference isolates found to belong to these taxa. In this context, it is encouraging that colour-group 4 is composed of *M. altitudinis* and *M. excelsi* strains and that colour-group 3 encompasses three of the four isolates shown to belong to the genus *Pseudonocardia*. However, most of the colour-groups encompass isolates that can be considered to be members of the genus *Streptomyces*. Several of the Atacama Desert isolates can be considered to represent putatively novel species, as shown by relationships between isolates 2SCA1, 3G5, 4SCA5, and 2G9 and the type strains of their close phylogenetic neighbours, namely *Kribbella turkmenica* (98.94% sequence similarity), *Pseudonocardia xinjiangensis* (97.81% sequence similarity), *Streptomyces camponoticapitis* (97.58% sequence similarity) and *Streptomyces paradoxus* (98.79% sequence similarity), respectively.

Using the approach outlined above isolates from the saline soil fell into two genera, *Nocadiopsis* (colour-groups 4 to 6 and 8) and *Streptomyces* (colour-groups 1 to 3 and 7). Isolates OF4 and OF6 (colour-group 4) can be provisionally identified as *N. metallicus* as they shared identical or almost identical 16S rRNA gene sequence similarities with the type strain of this species. In contrast, isolates IT2 and IF12, which represent single-membered colour-groups 6 and 5, are putatively novel *Nocardiopsis* species as they show sequence similarities of 97.45% and 98.72% with the type strains of *Nocardiopsis flavescens* and *Nocardiopsis halotolerans*, respectively. Similarly, isolate IF7 from colour-group 3 shared a 97.24% sequence similarity with *Streptomyces cahuitamycinicus* 13K301^T^. The two largest taxa, colour-groups 1 and 2, were composed of isolates which were found to have identical or very high sequence similarities with type strains of *Streptomyces alkaliterrae* and *Streptomyces alkaliphilus,* respectively.

The most pronounced taxonomic variation was found amongst the isolates from the litter and mineral layers of the northern slope of the inland pine dune. Following the procedure described above, colour-groups 7, 8, and 11 contain *Actinacidiphila* strains, colour-groups 4, 5, 13, 18, 19, and 21 *Streptomyces* strains whereas those in colour-groups 2 and 10 belong to the genus *Kitasatospora*. Similarly, isolates comprising colour-groups 1, 6, 12, 14, 15, 16, 17, and 20 can be considered to be members of the genus *Streptacidiphilus* and those forming colour-group 9 members of the genus *Pilimelia*. It is especially interesting that colour-group 3 encompasses nine isolates assigned to the genus *Catenulispora*, including the type strains of *C. pinisilvae* and *C. pinistramenti*. Several isolates, namely NA4, NL15, and NA21, can be considered as putatively novel species given low sequence similarities with their immediate phylogenetic neighbours, namely *Streptacidiphilus albus* NBRC 100918^T^ (97.73%), *Streptacidiphilus carbonis* DSM 41754^T^ (98.44%) and *Streptacidiphilis hamsterleyensis* (92.01%). Similarly, isolates NH28a, NL16, NL23, NF27, and NH28 are prospective novel species that are most closely related to the type strains of *Actinacidiphila alni* (98.50%), *Actinacidiphila bryophytorum* (98.48%), *Actinacidiphila paucisporea* (98.62%), *Actinacidiphila yanglinensis* (98.14%) and *Streptomyces paludis* (98.13%), respectively. Isolate NH17 shows a very low sequence similarity (92.32%) with its nearest neighbour, *Streptomyces luteireticuli* NBRC 13422^T^, suggesting that it may belong to a novel genus.

A different pattern of taxonomic diversity was found with strains from the litter and mineral horizons on the southern slope of the inland pine dune. Once again, many of the taxa contained isolates associated with the genus *Streptomyces*, as witnessed by colour-groups 1, 3, 4, 12, 13, 14, 15, 17, and 18 whereas those assigned to colour-groups 7, 8, 10, and 11 contained *Actinacidiphila* strains. Further, most, if not all, of the isolates comprising colour-groups 2 and 5 were closely related to the genus *Pilimelia* whereas those assigned to colour-groups 9 and 16 can be considered to be *Nocardia* and *Streptacidiphilus* strains, respectively. Several strains were found to belong to putative novel species, as exemplified by isolates SL52, SL13, SL5, SA10, SF9, SF13, and SL54, which shared low sequence similarities with the type strains of *A. bryophytorum* (98.61%), *Actinacidiphila rubida* (97.49%), *A. yanglinensis* (98.70%), *Actinospica acidiphila* (98.07%), *Nocardia nova* (98.85%), *Nocardia vinacea* (98.22%) and *Streptomyces ferralitis* (97.65%), respectively. Isolates related to the type strains of *A. bryophytorum, A. yanglinensis, P. columellifera* subspecies *pallida*, *Streptacidiphilus torunensis*, *Streptomyces atratus* and *S. celluloflavus* were also isolated from the northern inland pine dune.

### 3.4. Antimicrobial activity

Seventy-nine of the 115 isolates (69%) representing the colour-groups were active against at least one of the reference strains in the antimicrobial screening assay (Tables 2, 3). Those from the Atacama Desert soils were more active following growth on ISP3 than on ISP2 agar whereas the isolates from the saline soil showed more activity when grown on HA than on ISP3 agar. In contrast, the isolates from the pine forest soils tended to show similar patterns of activity irrespective of whether they were cultivated on ISP2 or ISP3 agar. Only strains from the pine forest soils inhibited the growth of the *K. pneumoniae* and *P. mirabilis* strains; they also showed more activity against *C. albicans* ATCC 10231 than those from the other sampling sites.

**Table 2:** Antimicrobial activity of representative actinomycetes isolated from the Atacama Desert and pine forest soils using a standard plug assay.

Strains	*B. subtilis* PCM 2021		*E. coli* PCM 2057		*K. pneumoniae* ATCC 700603		*M. luteus* ATCC 10240		*P. mirabilis* (CM NCU)		*P. aeruginosa* ATCC 10145		*S. infantis* (SES)		*S. aureus* PCM 2054		*C. albicans* ATCC 10231	
Basal medium	ISP2	ISP3	ISP2	ISP3	ISP2	ISP3	ISP2	ISP3	ISP2	ISP3	ISP2	ISP3	ISP2	ISP3	ISP2	ISP3	ISP2	ISP3
Representative isolates from the hyper-arid Atacama Desert soils																		
1G2	–	–	–	–	–	–	–	–	–	–	–	5.0* ±0.0	–	–	–	–	–	–
1G6^T^	–	–	–	–	–	–	2.0 ± 0.0	–	–	–	–	–	–	–	–	–	–	–
1G50	–	–	–	–	–	–	4.0* ±0.0	–	–	–	–	–	–	–	–	–	–	–
1HA1	–	–	–	–	–	–	–	–	–	–	–	–	–	–	2.0* ±0.0	–	–	–
1SCA19	–	3.0 ± 0.0	2.0 ± 0.0	–	–	–	7.0 ± 0.0	9.0* ±0.0	–	–	–	–	–	2.0 ± 0.0	2.0 ± 0.0	5.0 ± 0.0	–	–
1SCA21	–	–	–	–	–	–	5.7 ± 0.6	9.0 ± 0.0	–	–	–	–	–	3.0 ± 0.0	–	4.0* ±0.0	2.0 ± 0.0	–
2G9	2.0 ± 0.0	5.0 ± 0.0	–	–	–	–	10.0 ± 0.0	13.0 ± 0.0	–	–	–	–	–	–	4.0 ± 0.0	4.0 ± 0.0	–	–
3HA10	–	9.0 ± 0.0	–	–	–	–	–	6.0 ± 0.0	–	–	–	–	–	–	2.0* ±0.0	4.0 ± 0.0	–	15.0 ± 0.0
4R2A1	–	–	–	–	–	–	–	–	–	–	–	–	–	–	2.0 ± 0.0	–	–	–
4SCA5	–	2.3 ± 0.6	–	–	–	–	7.0 ± 0.0	10.0 ± 0.0	–	–	–	–	–	–	–	3.3 ± 0.6	–	–
5HA2	2.0* ±0.0	4.0 ± 0.0	–	–	–	–	5.0 ± 0.0	4.3 ± 0.6	–	–	–	–	–	–	–	–	–	–
5R2A3	–	–	–	–	–	–	–	–	–	–	–	–	–	–	–	–	10.0 ± 0.0	–
5R2A7^T^	–	–	–	–	–	–	–	2.0 ± 0.0	–	–	–	–	–	–	–	–	–	–
5SCA4	–	5.0 ± 0.0	–	–	–	–	–	2.0 ± 0.0	–	–	–	–	–	–	–	–	–	–
5SCA5	4.0 ± 0.0	3.0 ± 0.0	–	–	–	–	–	–	–	–	–	–	–	–	6.0 ± 0.0	–	–	–
5SCA7	3.0 ± 0.0	–	–	–	–	–	–	–	–	–	–	–	–	–	6.0 ± 0.0	–	–	–
Representative isolates from the litter and mineral horizons of the northern slope of the inland dune of the pine forest																		
NA4	–	–	–	–	–	–	–	–	–	–	–	–	–	–	–	–	2.0 ± 0.0	11.0 ± 0.0
NA10a	4.0 ± 0.0	7.0* ±0.0	2.0 ± 0.0	3.0 ± 0.0 4.0* ±0.0	–	–	5.0 ± 0.0	13.0 ± 0.0	–	2.0 3.0* ±0.0	–	–	–	2.0 ± 0.0	2.0 ± 0.0	4.0 ± 0.0	12.0 ± 0.0	14.0 ± 0.0
NA13	8.0 ± 0.0	–	–	–	–	–	3.0 ± 0.0	–	–	–	–	–	–	–	–	–	–	–
NA21	–	–	5.0 ± 0.0	–	2.0 ± 0.0	–	–	–	–	–	–	–	12.0 ± 3.6	–	–	–	9.3 ± 0.6	10.0 ± 0.0
NA24	–	8.0 ± 0.0	–	–	–	–	14.3 ± 0.6	10.0 ± 0.0	–	–	–	–	–	–	10.0 ± 2.0	8.0 ± 0.0	7.7 ± 0.6	10.0* ±0.0
NF3	–	–	–	–	–	–	–	2.0* ±0.0	–	–	–	–	–	2.0 ± 0.0	–	–	–	4.0* ±0.0
NF22	–	–	–	4.0 ± 0.0	–	–	–	–	–	–	–	–	–	–	–	–	–	13.0 ± 0.0
NF24	–	–	–	–	–	–	–	–	–	–	–	–	–	–	–	–	–	2.0* ±0.0
NF39	–	–	–	3.0 ± 0.0	–	–	–	–	–	–	–	–	–	–	–	–	–	–
NH7	2.0 ± 0.0	9.0* ±0.0	–	4.0 2.0* ±0.0	–	–	4.0 ± 0.0	14.0 ± 0.0	–	6.0 ± 0.0	–	–	–	3.0 ± 0.0	–	5.0 ± 0.0	–	15.0 ± 0.0
NH9	–	–	–	–	–	–	–	–	–	–	–	–	–	–	–	–	–	3.0* ±0.0
NH11^T^	5.0 ± 0.0	–	–	–	–	–	8.7 ± 1.6	2.0 ± 0.0	–	–	–	–	–	–	–	–	–	3.0* ±0.0
NH16	–	–	–	2.0* ±0.0	–	–	–	5.0 ± 0.0	–	–	–	–	–	–	–	–	–	–
NH17	–	4.0 ± 0.0	5.7 ± 0.6	–	–	–	8.7 ± 0.6	11.0 ± 0.0	–	–	–	–	–	3.0 ± 0.0	–	6.0 ± 0.0	9.3 ± 0.6	12.0 ± 0.0
NH21	–	–	–	–	2.0 ± 0.0	–	–	–	–	–	–	–	–	–	–	–	–	–
NH22	–	–	–	2.0* ±0.0	–	–	–	–	–	–	–	–	–	–	–	–	15.0 ± 0.0	8.0 ± 0.0
NH27	–	–	–	–	–	–	–	–	–	–	–	–	–	–	–	–	–	–
NH28	1.3 ± 0.6	–	–	–	2.0 ± 0.0	–	6.7 ± 0.6	10.0 ± 0.0	–	–	–	–	–	2.0 ± 0.0	–	4.0* ±0.0	7.3 ± 0.6	9.0 ± 0.0
NH28a	9.0 ± 0.0	9.0 ± 0.0	–	–	–	–	11.7 ± 0.6	8.0 ± 0.0	–	–	–	–	15.3 ± 5.0	–	9.0 ± 0.0	7.0 ± 0.0	–	–
NL8^T^	–	–	–	2.0* ±0.0	–	–	5.0 ± 0.0	–	–	–	–	–	–	–	–	–	–	–
NL15	–	–	–	–	2.0 ± 0.0	–	3.3 ± 1.2	–	–	–	–	–	2.0* ±0.0	–	–	–	–	–
NL16	–	–	–	2.0* ± 0.0	–	–	–	5.0 ± 0.0	–	–	–	–	–	–	–	–	–	–
NL21	12.0 ± 0.0	8.0 ± 0.0	–	–	7.7 ± 0.6	–	13.7 ± 0.6	3.0 ± 0.0	–	–	–	–	2.0* ±0.0	3.0 ± 0.0	12.0 ± 1.0	8.0 ± 0.0	9.7 ± 0.6	9.0* ±0.0
NL23	–	–	–	2.0* ± 0.0	–	–	–	–	–	–	–	–	–	–	–	–	2.0 ± 0.0	11.0 ± 0.0
NL28	–	–	–	–	–	–	–	5.0 ± 0.0	–	2.0 3.0* ±0.0	–	–	–	2.0 ± 0.0	2.0 ± 0.0	4.0 ± 0.0	12.0 ± 0.0	14.0 ± 0.0
Representative isolates from the litter and mineral horizons of the southern slope of the inland dune of the pine forest																		
SA4	14.0 ± 1.0	–	7.0 ± 0.6	–	4.3 ± 0.6	5.0* ±0.0	3.7 ± 0.6	–	7.7 ± 0.6	–	2.7 ± 0.6	–	4.3 ± 0.6	–	6.0 ± 0.0	5.0* ±0.0	–	–
SA7	–	–	3.3 ± 0.6	2.0* ±0.0	3.3 ± 0.6	7.0 ± 0.0	3.7 ± 0.6	14.0* ±0.0	–	–	2.3 ± 0.6	–	3.3 ± 0.6	4.0 ± 0.0	4.7 ± 1.2	7.0 ± 0.0	–	5.0 ± 0.0
SA10	–	8.0* ± 0.0	–	–	–	–	–	–	–	–	–	–	–	–	–	–	–	–
SA16	–	–	–	–	–	7.0* ±0.0	–	–	–	–	–	–	–	–	–	7.0* ±0.0	–	–
SF4	12.0 ± 0.0	–	–	–	3.3 ± 1.2	–	3.3 ± 0.6	–	–	–	–	–	–	–	14.3 ± 0.6	7.0 ± 0.0	–	–
SF8	–	–	3.7 ± 0.6	4.0 ± 0.0	3.0 ± 0.0	4.0 ± 0.0	6.0 ± 0.0	11.0* ±0.0	–	–	2.7 ± 0.6	–	3.0 ± 0.0	3.0 ± 0.0	4.7 ± 0.6	5.0 ± 0.0	9.7 ± 0.6	5.0 ± 0.0
SF10	–	–	–	–	–	–	–	–	–	2.0* ±0.0	–	–	–	–	7.0 ± 0.0	–	–	–
SF13	–	–	–	–	–	–	–	–	–	2.0* ±0.0	–	–	–	–	–	–	–	–
SF15	10.7 ± 0.6	4.0 ± 0.0	4.7 ± 0.6	–	4.3 ± 0.6	7.0* ±0.0	4.3 ± 0.6	–	–	–	2.0 ± 0.0	–	3.3 ± 1.2	–	7.7 ± 0.6	7.0* ±0.0	–	–
SF17	–	–	–	–	–	5.0* ±0.0	–	–	–	–	–	–	–	–	7.0 ± 1.0	5.0* ±0.0	–	–
SF28^T^ **	9.7 ± 1.2	–	3.0 ± 1.0	–	2.0 ± 0.0	–	3.0 ± 0.1	–	–	–	3.0 ± 0.1	–	3.3* ±0.6	–	6.2 ± 0.6	–	–	–
SH11	10.3 ± 1.2	–	3.0 ± 1.0	5.0 ± 0.0	2.3 ± 0.6	7.0 ± 0.0	4.0 ± 0.0	13.0* ± 0.0	–	6.0 ± 0.0	2.3 ± 0.6	–	3.0 ± 1.0	–	4.3 ± 0.6	7.0 ± 0.0	10.0 ± 0.0	7.0 ± 0.0
SH15	3.0 ± 0.0	7.7 ± 0.6	–	3.0 5.0* ±0.0	–	5.0* ±0.0	5.0 ± 0.0	12.7 ± 0.6	–	3.0 ± 0.0	–	–	–	3.0 ± 0.0	–	5.0* ±0.0	10.0 ± 0.0	15.0 ± 0.0
SH20	–	–	–	–	–	6.0 ± 0.0	–		–	–	–	–	–	–	–	6.0 ± 0.0	–	–
SH24	11.0 ± 1.0	–	5.3 ± 1.2	–	4.0 ± 0.0		3.3 ± 0.6		8.3 ± 1.5	–	2.3 ± 0.6	–	4.3 ± 0.6	4.0 ± 0.0	7.3 ± 1.2	–	–	–
SH56	–	–	–	–	–	–	2.0* ±0.0	7.0* ±0.0	4.0 ± 0.0	–	–	–	8.0 ± 1.2	2.0* ±0.0	–	–	3.7 ± 0.6	–
SH57	2.0* ±0.0	–	–	–	–	–	2.3 ± 0.6	2.0* ±0.0	–	–	–	–	–	–	–	–	–	10.0 ± 0.0
SL3	2.0 ± 0.0	–	4.0* ±0.0	3.0 4.0* ±0.0	–	–	5.0 ± 0.0	13.0 ± 0.0	–	5.0 ± 0.0	–	–	–	3.0 ± 0.0	–	4.0* ±0.0	13.0 ± 0.0	13.0 ± 0.0
SL4	10.0 ± 0.0	–	–	–	–	–	2.7 ± 0.6	–	–	–	–	–	–	–	13.3 ± 0.6	9.0 ± 0.0	–	–
SL5	5.0 ± 0.0	3.0 ± 0.0	–	–	–	–	6.0 ± 0.0	6.0 ± 0.0	–	–	–	–	–	–	5.0 ± 0.0	3.0 ± 0.0	–	–
SL7	4.0* ±0.0	5.0 ± 0.0	–	–	6.0* ± 0.0	–	4.0 ± 0.0	5.0 ± 0.0	–	–	–	–	–	–	4.0* ±0.0	2.0 ± 0.0	6.0 ± 0.0	6.0 ± 0.0
SL10	9.0 ± 0.0	–	3.0 ± 0.0	–	–	–	7.3 ± 1.2	6.0 ± 0.0	–	–	–	–	–	–	9.0 ± 0.0	5.0 ± 0.0	–	–
SL19	–	8.0 ± 0.0	–	–	–	–	–	9.0 ± 0.0	–	–	–	–	–	–	–	8.0 ± 0.0	–	–
SL22	5.0 ± 0.0	6.7 ± 0.6	–	–	–	–	6.0 ± 0.0	10.0 ± 0.0	–	–	–	–	–	–	5.0 ± 0.0	5.0 ± 0.0	–	–
SL24	–	–	–	4.0* ±0.0	–	8.0 ± 0.0	–	–	–	–	–	–	–	6.0* ±0.0	–	–	–	–
SL55	3.0* ±0.0	2.0 ± 0.0	4.0 ± 0.0	–	–	2.0* ±0.0	–	–	–	–	–	–	–	–	8.0 ± 0.0	2.0* ±0.0	–	–

Inhibition zones of growth were measured in mm, the values are the mean of three replicates ± standard deviations.

–, no inhibition; *isolates showing poor growth; **Antimicrobial activity of *C. pinisilvae* NH11^T^ and NF3, and *S. pinistramenti* SF28^T^ was mentioned previously (Świecimska et al., 2021a, 2022). ^T^, type strain.

Isolates which did not inhibit the growth of any of the strains: (A) Atacama Desert: 1G4^T^, 1G14, 1G51, 1G52, 1HA3, 1R2A1, 1R2A7, 2G7, 2R2A4, 2SCA1, 3G5, and 3G6; (B) northern slope of the inland dune: NA19a, NF10, NF20, NF23, NF27, NH5, NH14, NH15, NL3, NL13, and NL35; (C) southern slope of the inland dune: SA8, SA20, SA23, SF9, SF23, SL13, SL16, SL52, and SL54.

**Table 3:** Antimicrobial activity of isolates from the saline soil adjacent to Lake Lonar using a standard plug assay.

Strain	*B. subtilis* PCM 2021		*M. luteus* ATCC 10240		*P. aeruginosa* ATCC 10145		*S. infantis* (SES)		*C. albicans* ATCC 10231	
	HA	ISP3	HA	ISP3	HA	ISP3	HA	ISP3	HA	ISP3
IF7	–	–	5.3 ± 0.6	–	–	–	–	–	–	–
IF11	–	–	2.3 ± 2.1	–	–	–	–	–	–	–
IF15	–	–	–	–	–	–	–	2.0* ±0.0	–	–
IT2	3.0 ± 0.0	–	–	–	–	–	–	–	–	–
OF1^T^	–	–	8.3 ± 0.6	–	2.0 ± 0.0	–	–	–	2.0 ± 0.0	–
OF2	–	–	2.7 ± 2.5	13.0 ± 0.0	2.3 ± 0.6	–	–	–	2.0 ± 0.0	–
OF3	–	–	–	14.0 ± 0.0	–	–	–	–	2.7 ± 0.6	–
OF5	–	–	5.0 ± 0.0	–	4.0 ± 0.0	–	–	–	2.7 ± 0.6	–
OF6	–	–	4.0 ± 0.0	–	–	–	–	–	–	–
OF7	–	–	17.3 ± 0.6	12.0 ± 0.0	3.0 ± 0.0	–	–	–	2.0 ± 0.0	4.0 ± 0.0
OF8	–	–	15.7 ± 0.6	11.0 ± 0.0	2.0 ± 0.0	–	–	–	3.0 ± 0.0	–
OT1	–	–	4.3 ± 1.2	–	–	–	–	–	–	–

Inhibition zones of growth were measured in mm, the values are the mean of three replicates ± standard deviations.

–, no inhibition; *isolates showing poor growth; Isolates IF12, IF17, IF19, and OF4 did not inhibit the growth of any of the strains.

None of the isolates, irrespective of whether they were cultivated on HA or ISP3 media inhibited the growth of *E. coli* PCM 2057, *K. pneumoniae* ATCC 700603, *P. mirabilis* (CM NCU) or *S. aureus* PCM 2054.

Table 2A shows that the Atacama Desert isolates were most active against the Gram-positive reference strains. In contrast, only isolate 1SCA19, which is most closely related to *Streptomyces purpurascens* NBRC 13077^T^ (sequence similarity 99.15%), and isolates 5R2A3 and 1SCA21 inhibited the growth of the *E. coli* and *C. albicans* strains following cultivation on ISP2 agar. The most pronounced activity was shown by isolates 2G9, 3HA10, and 1SCA19 which are, in turn, most closely related to *Streptomyces paradoxus* NBRC 14887^T^ (98.79% sequence similarity), *Streptomyces galbus* DSM 40089^T^ (99.37% sequence similarity) and the type strain of *Streptomyces purpurascens* (99.15% sequence similarity), respectively. Twelve of the isolates from the hyper-arid Atacama Desert soils, namely ones belonging to the genera *Kribbella* (isolates 2G7 and 2SCA1), *Modestobacter* (isolates 1G4^T^, 1G51, 1G52, and 1G14), *Pseudonocardia* (isolates 1HA3, 2R2A4, and 3G5) and *Streptomyces* (isolates 1R2A7, 3G6, and 1R2A1) did not inhibit the growth of any of the reference strains.

The most active strains from the northern slope of the inland pine dune were found to inhibit the growth of the *C. albicans, E. coli*, *M. luteus* and *S. infantis* reference strains. Isolates NA10a, NA24, NH7, NH17, NH28, and NL21 were particularly active against *C. albicans* ATCC 10231 and *M. luteus* ATCC 10240 (inhibition zones 3.0–15.0 mm; Table 2B); isolates NA10a and NH7, and NA24 and NL21 were most closely related to the type strains of *S. celluloflavus* and *S. xanthochormogenes*, respectively, whereas isolates NH17 and NH28 are members of putatively novel *Streptomyces* species (Table 1). Other strains showing activity against the *C. albicans* strain (inhibition zones 2.0–15.0 mm) were isolates NA4, NA21, prospective members of novel *Streptacidiphilus* species, isolate NH22, which shares a 99.78% sequence similarity with *Streptacidiphilus neutrinimicus* DSM 41755^T^, and NL23, a prospective novel *Actinacidiphila* isolates most closely related to *A. paucisporea* CGMCC 4.2025^T^. Similarly, *C. pinisilvae* NH11^T^ and isolate NH28a, which are also members of putatively novel *Actinacidiphila* and *Streptomyces* species, respectively, showed pronounced activity against the *M. luteus* strain; isolate NH28a also inhibited the growth of the *S. infantis* strain following growth on ISP2 agar, as did isolate NA21, a representative of a prospective novel *Streptacidiphilus* species.

The most active isolates from the northern slope of the inland pine dune, isolates NA10a and NH7, close relatives of the type strain of *S. celluloflavus*, inhibited the growth of seven of the reference strains following cultivation on either ISP2 or ISP3 agar. Eleven isolates inhibited the growth of *E. coli* PCM 2057 following cultivation on one or both of the cultivation media, namely isolates NA10a, NA21, NH7, and NH17 mentioned above, *C. pinistramenti* NL8^T^, and isolates NF22, NH22, NF39, and NH16, which are most closely related to the type strains of *S. hamsterleyensis*, *S. neutrinimicus*, *K. herbaricolor* and *S. atratus*, respectively, and isolates NL16 and NL23, prospective members of novel *Actinacidiphila* species (Table 1). Five strains inhibited the growth of *K. pneumoniae* ATCC 700603 following growth on ISP2 agar, namely isolate NA21 mentioned above, isolates NH21 and NL21, which share sequence similarities of 99.07% and 99.93% with *A. yanglinensis* 1307^T^ and *S. xanthochromogenes,* NRRL B-5410^T^, respectively, and isolates NL15 and NH28, presumptive members of novel *Streptacidiphilus* and *Streptomyces* species, respectively. Nine isolates inhibited the growth of *B. subtilis* PCM 2021, as shown in Table 2B. In contrast, the 11 isolates which did not inhibit the growth of any of the reference strains were members of the genera *Actinacidiphila* (isolate NF27), *Catenulispora* (isolates NF23 and NL13), *Streptacidiphilus* (isolates NA19a, NF10, NF20, and NH14) and *Streptomyces* (isolates NH5, NH15, NL3, and NL35).

The isolates from the corresponding southern slope of the pine forest exhibited a different pattern of activity to their counterparts from the northern slope (Table 2). Thirteen isolates, for instance, inhibited the growth of the *S. aureus* PCM 2054, namely isolate SF17, a close relative of *A. yanglinensis* 1307^T^, isolates SA4, SF15, and SL4, which showed their highest sequence similarities with *P. columellifera* subsp. *pallida* MB-SK8^T^, isolates SA7, SF4, SF8, and SH11, close relatives of *S. celluloflavus* NRRL B-2493^T^, isolates SL5, SL7, SL10, and SL22, which are prospective novel members of *Actinacidiphila* species, and isolate SL55, a prospective member of a novel *Pilimelia* species (Table 1D) following growth on ISP2 and ISP3 media. Eleven isolates inhibited the growth of the *M. luteus* reference strain, namely isolates SA7, SF8, SH11, SL3, SL5, SL7, SL10, and SL22 mentioned above, isolate SH15, a close relative of *S. celluloflavus* NRRL B-2493^T^ and isolates SH56 and SH57 which are close to *Streptomyces cocklensis* BK 168^T^ and *Streptomyces halstedii* NBRC 12783^T^, as shown in Table 1. *Bacillus subtilis* PCM 2021 was inhibited by isolates SF15, SH15, SL5, SL7, SL22, and SL55 mentioned above whereas *S. infantis* SES was inhibited by isolates SA7, SF8, and SH56 mentioned earlier, and isolate SH24, a close relative of *P. columellifera* subspecies *pallida* (inhibition zones ranging from 2.0 to 8.0 mm) following growth on ISP2 and ISP3 agar.

Many of the isolates from the southern slope of the pine forest dune inhibited the growth of the Gram-negative reference strains. Fifteen of them were active against *K. pneumoniae* ATCC 700603 (43%), the corresponding numbers for *E. coli* PCM 2057, *S. infantis* SES, *P. mirabilis* CM NCU and *P. aeruginosa* ATCC 10145 were found to be 12 (34%), 11 (31%), 8 (23%), and 7 (20%), respectively. Four of the eight isolates shown to be closely related to *P. columellifera* subspecies *pallida* MB-SK8^T^ (isolates SA4, SF15, SH24, and SL24) inhibited the growth of the *E. coli* strain (inhibition zones 4.0–7.0 mm), as did *S. pinistramenti* SF28^T^, isolate SL10, a prospective member of a new *Actinacidiphila* species most closely related to the type strain of *A. yanglinensis* and isolate SL55, a prospective member of a novel *Pilimelia* species. Similarly, four of the six isolates closely related to *S. celluloflavus* NRRL B-2493^T^ (isolates SA7, SF8, SH11, and SL3) suppressed the growth of the *E. coli* strain (inhibition zones 2.0–5.0 mm) following growth on both cultivation media whilst isolate SH15, one of the two remaining *S. celluloflavus* isolates, showed similar activity following growth on ISP3 agar.

Five of the 15 isolates cultivated on ISP2 or ISP3 that were active against *K. pneumoniae* ATCC 700603 were close relatives of either *P. columellifera* subspecies *pallida* MB-SK8^T^ (isolates SA4 and SF15) or *S. celluloflavus* NRRL B-2493^T^ (isolates SA7, SF8, and SH11). The 10 isolates which inhibited the growth of this reference strain following growth on either ISP2 or ISP3 agar were isolates SH24 and SL24, and SF4 and SH15, close relatives of the type strains of *P. columellifera* subspecies *pallida* and *S. celluloflavus*, respectively, *S. pinistramenti* SF28^T^, isolates SF17 and SA16, which are most closely related to *A. yanglinensis* and *S. sanglieri*, and isolates SL7, SH20, and SL55, which were shown to be members of putatively novel *Actinacidiphila*, *Nocardia* and *Pilimelia* species, respectively, as shown in Table 1. Similarly, the eight strains that inhibited the growth of *P. mirabilis* CM NCU included five related to the type strains of *P. columellifera* subspecies *pallida* (isolates SA4 and SH24) and *S. celluloflavus* (isolates SH11, SH15, and SL3), isolate SF13, a presumptively novel *Nocardia* species, and isolates SF10 and SH56 which share 100% and 99.15% sequence similarity to *Streptacidiphilus torunensis* NF37^T^ and *Streptomyces cocklensis* BK168^T^, respectively. The seven strains active against the *P. aeruginosa* strain (inhibition zones 2.0–3.0 mm) were *S. pinistramenti* SF28^T^ and ones closely related to either the type strains of *P. columellifera* subspecies *pallida* (isolates SA4, SF15, and SH24) or *S. celluloflavus* (isolates SA7, SF8, and SH11). Four of the strains mentioned above, isolates SA7, SF8, SH24, and SH56, inhibited the growth of the *S. infantis* strain (inhibition zones ranging from 2.0 to 8.0 mm). In turn, several isolates inhibited the growth of *C. albicans* ATCC 10231, notably isolates SF8, SH11, SH15, SL3, and SL7 which gave inhibition zones ranging from 5.0 to 15.0 mm. The nine isolates which did not show any activity against the reference strains belonged to the genera *Actinacidiphila* (isolates SA23, SL13, and SL52), *Nocardia* (isolate SF9), *Pilimelia* (isolates SF23 and SL16) and *Streptomyces* (isolates SA8, SA20, and SL54).

Table 3 shows that some of the isolates from the saline soil inhibited the growth of the *B. subtilis, C. albicans, M. luteus, P. aeruginosa* and *S. infantis* strains. In contrast, none of them were active against *E. coli* PCM 2057, *K. pneumoniae* ATCC 700603, *P. mirabilis* CM NCU or *S. aureus* PCM 2054. It is noteworthy that all of the *S. alkaliterrae* isolates, apart from OF3, inhibited the growth of *P. aeruginosa* ATCC 10145 and *M. luteus* ATCC 10240 following growth on HA agar. Isolate IT2 was the only *Nocardiopsis* strain to show any activity; it inhibited the growth of the *B. subtilis* strain and may represent a novel species as it was only loosely associated with its closest phylogenetic neighbour, *Nocardipsis flavescens* CGMCC 4.5723^T^ (97.45% sequence similarity).

### 3.5. Plant growth promoting features

**Figure 1:**
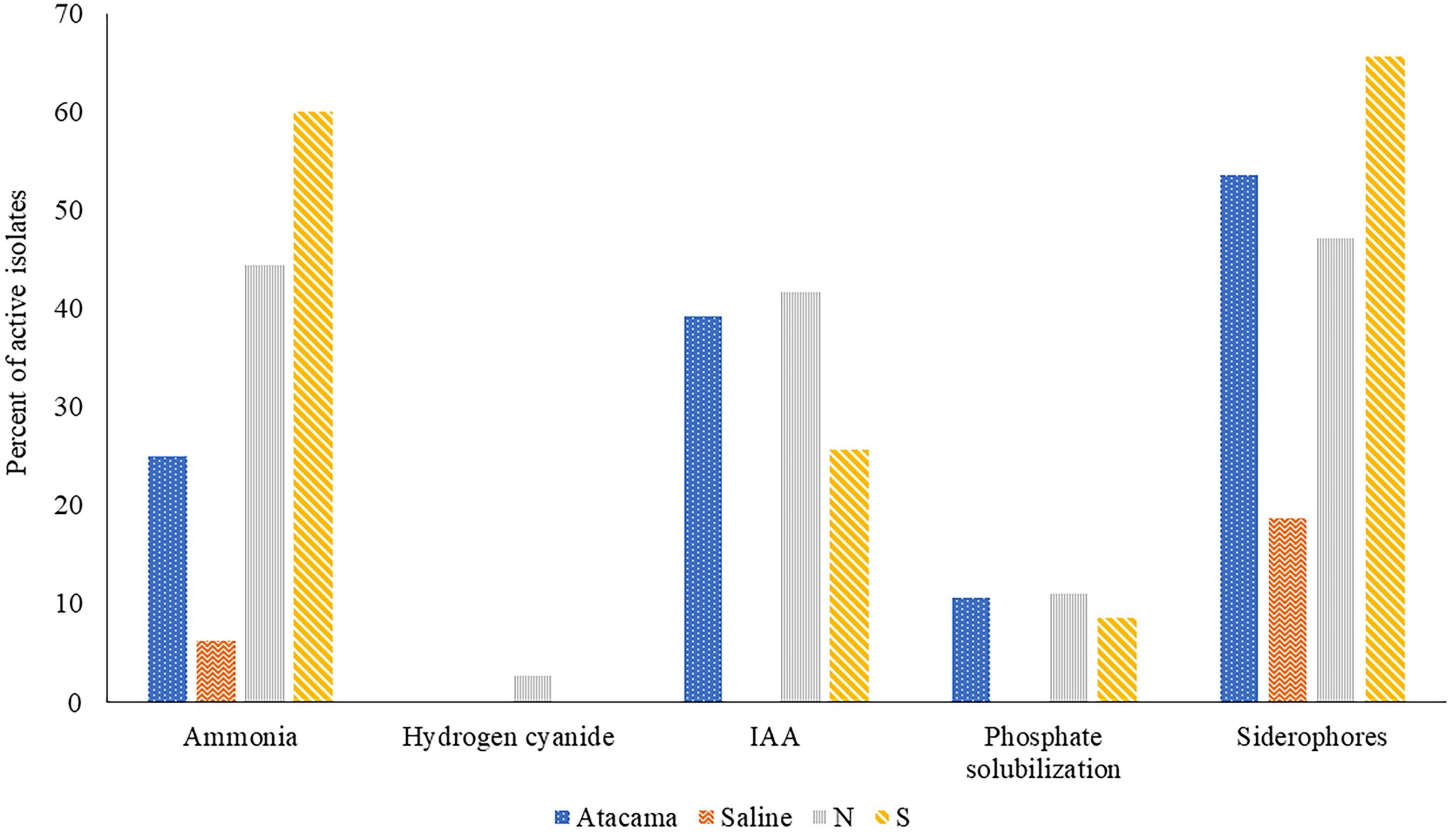
Representative isolates [%] from the Atacama Desert, saline and acid forest soil sampling sites which were found to produce compounds that promote plant growth. N and S, isolates from the northern and southern slopes of the inland dunes of the pine forest.

The results of the triplicated analyses designed to determine the ability of representatives of the colour-groups to produce plant growth promoting compounds are shown in Figure 1 and Supplementary Table S3. It is apparent from the Figure 1 that many of the isolates from the Atacama Desert and pine forest samples produced ammonia, IAA and siderophores, but relatively few solubilised phosphate. In contrast, none of the isolates from the saline soil produced IAA or were active in solubilising phosphate whilst few produced ammonia or siderophores, *Catenulispora pinistramenti* NL8^T^ was the only isolate found to produce hydrogen cyanide (HCN), a volatile compound that has a role in biocontrol by sequestering iron at the expense of phytopathogens (Gu et al., 2020).

Seven isolates from the Atacama Desert soils (25%) showed an ability to release ammonia whilst the corresponding figures for those from the saline soil and from northern and southern inland pine slopes were 1 (6%), 16 (44%) and 21 (60%), respectively. The most active strains were *S. pinistramenti* SF28^T^, isolates NA10a and SA16, near phylogenetic neighbours to the type strains of *S. celluloflavus* and *S. sanglieri*, respectively, and isolates NL15 and NH28, which were found to be members of prospective novel species of *Streptacidiphilus* and *Streptomyces,* as they share low sequence similarities with their close phylogenetic relatives, *S. carbonis* DSM 41954^T^ (98.44% sequence similarity) and *S. paludis* GSSD-12^T^ (98.13% sequence similarity), respectively. Strains showing a less pronounced ability to form ammonia included isolates NL3 and SA7 which are close to *S. celluloflavus* NRRL B-2493^T^, isolates NA24 and NL21, which have a similar relationship to *S. xanthochromogenes* NRLL B-5410^T^, and isolates NH9, NA19a, and NL35, which are close to *K. herbaricolor* (99.43% sequence similarity), *Streptacidiphillus durhamensis* (99.86% sequence similarity) and *Streptomyces yanii* (99.12% sequence similarity), respectively. Isolate OT1, the only strain from the saline soil to produce ammonia, was found to share a sequence similarity of 99.50% with its immediate phylogenetic neighbour, *Nocardiopsis valliformis* DSM 45023^T^.

It is evident from Supplementary Table S2 that 11 isolates (39%) from the Atacama Desert soils synthesised IAA, the corresponding numbers from the northern and southern slopes of the pine forest were 42% and 26%, respectively. The most active strains were isolates 1HA1, 5R2A3, and SA8, which have identical or almost identical sequence similarities with *K. italica* BC637^T^, *M. acroterricola* 5R2A7^T^ and *S. sanglieri* NBRC 100784^T^, as shown in Table 1. Twelve strains were less active in synthesising IAA, including *C. pinisilvae* NH11^T^. *Catenulispora pinistramenti* NL8^T^, isolates NF10 and NF20, which share identical or almost identical sequence similarities with *S. torunensis* NF37^T^, and isolate 1SCA21 mentioned earlier. This group of isolates can be extended to include NL16 and NF27, members of prospective novel *Actinacidiphila* species that are most closely related to *A. bryophytorum* NEAU-HZ10^T^ (98.48% sequence similarity) and *A. yanglinensis* 1307^T^ (98.14% sequence similarity), respectively.

Fifty-eight isolates (50%) produced siderophores with activity indices ranging from 0.3 to 17.7. Fifteen of those from the Atacama Desert (54%) showed positive activity indices whereas only three of the isolates from the saline soil (19%) did so; the corresponding numbers for those from the litter and mineral horizons of the northern and southern slopes of the inland pine dunes were 17 (47%) and 23 (66%), respectively. The six Atacama Desert strains with the highest activity indices (range 9.3–17.7) were isolates 1G2 and 1R2A1, close relatives to *S. flaveolus* NBRC 3715^T^ (99.72% sequence similarities), isolate 1SCA21 mentioned above, isolates 5SCA7 and 5HA2, near relatives to *Streptomyces marokkonensis* Ap1^T^ (99.50% sequence similarity) and *S. mutabilis* NBRC 12800^T^ (99.79% sequence similarity), respectively, and isolate 2G9, which is close to *S. paradoxus* NBRC 14887^T^. Two of the three active isolates from the saline soil, OF5 and IF17, were found to be phylogenetically close to *S. alkaliterrae* and *S. alkaliphilu*s, respectively; the remaining strain, isolate IF7, as mentioned previously, represent a presumptively novel *Streptomyces* species. The seven isolates from the pine forest sampling sites which gave the highest activity indices (range 8.6–14.2) were of *C. pinisilvae* NF3, *S. pinistramenti* SF28^T^, isolates NH7, SA7 and SH11, and isolate SH57, close relatives of *S. celluloflavus* NRRL 2493^T^, *S. halstedii* NBRC 12783^T^, respectively, and isolate NH17, a member of a prospective novel *Streptomyces* species. Similarly, five of the remaining six strains close to the type strain of *S. celluloflavus*, that is, isolates NA10a, SF4, SF8, SH15, and SL3, were found to have activity indices within the range 1.3–6.6. Similarly, isolates NF24, NF39 and NH9, phylogenetic neighbours to *K. herbaricolor* NBRC 12876^T^, have activity indices ranging from 2.8 to 4.1.

Few isolates from the Atacama Desert and pine forest sites solubilised phosphate (Supplementary Table S3). The three active isolates from the desert soils were 5R2A3, 1R2A7, and 1SCA21, near relatives of *M. acroterricola* 5R2A7^T^, *S. aquilus* GGCR-6^T^ and *S. tendae* ATCC 19812^T^, respectively. The isolates from the pine forest sites which solubilised phosphate were isolates NF24, NF39 and NH9, and SF8, SH15, and SL3, which are closely related to the type strains of *K. herbaricolor* and *S. celluloflavus*, respectively, and isolate NL21, a close relative to *S. xanthochromogenes* NRRL B-5410^T^.

### 3.6. Enzyme activity

**Figure 2:**
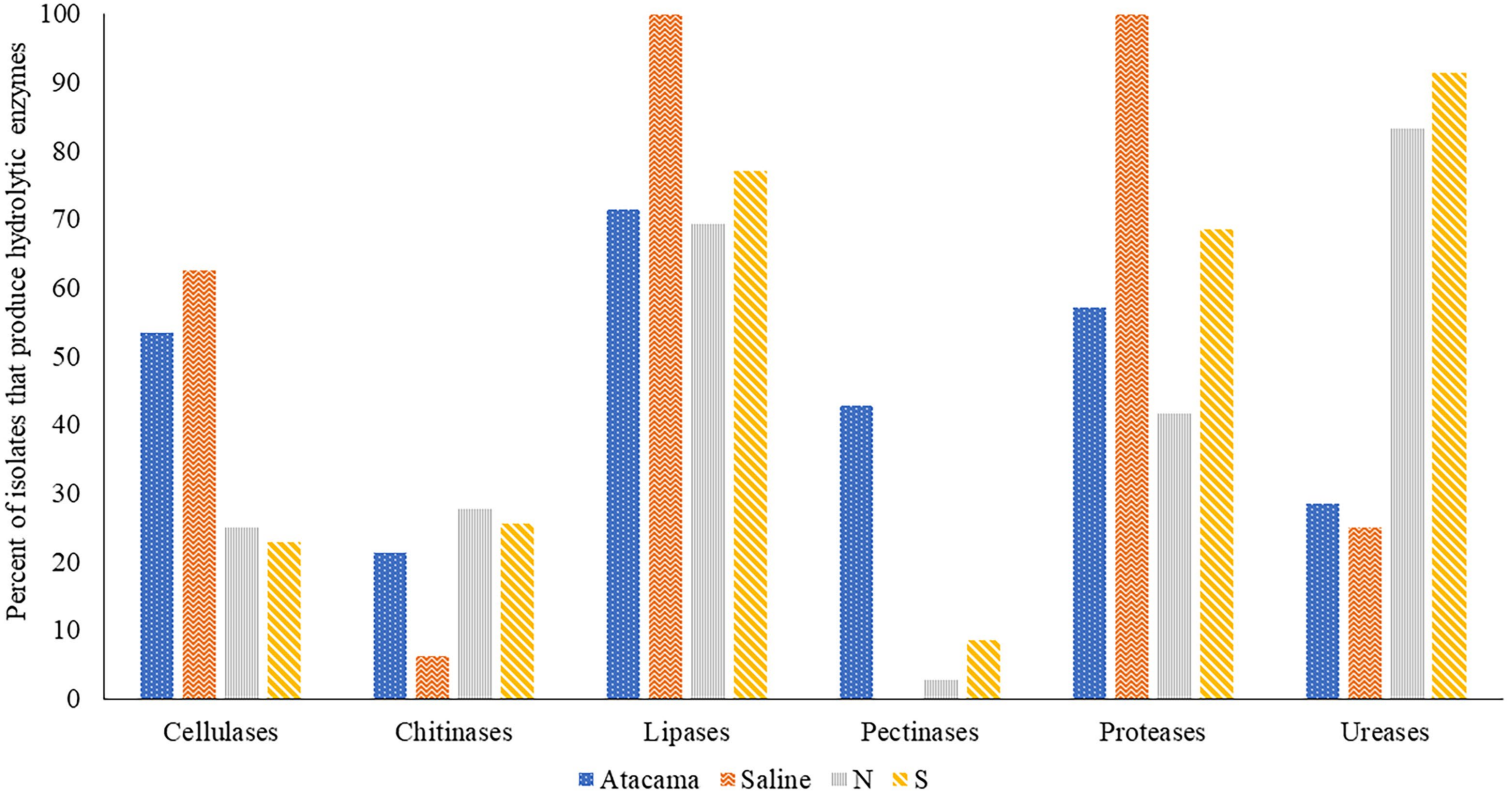
Representative isolates [%] from the Atacama Desert, saline and acidic pine forest soil which were found to produce hydrolytic enzymes. N and S, isolates from the northern and southern slopes of the inland dunes of pine forest.

The ability of isolates representing the colour-groups to synthesise hydrolytic enzymes is shown in Figure 2 and Supplementary Table S3. It is evident from the Figure that many of the isolates from each of the sampling sites produce cellulases, lipases, proteases and ureases, and to a lesser extent chitinases. All of the isolates from the saline soil hydrolysed tributyrin and showed proteolytic activity, and most from the pine forest sites hydrolysed urea. In contrast, only isolates from the Atacama Desert and pine forest soils hydrolyzed pectin. Eighty-eight of the isolates (77%) produced zones of clearing against tributyrin; the corresponding results for the hydrolysis of cellulase, chitin, milk powder, pectin and urea were 42 (37%), 26 (23%), 71 (62%), 16 (14%) and 74 (64%), respectively. In contrast, pectinases were mainly produced by isolates from the Atacama Desert soils. The isolates from the saline soil metabolised tributyrin, but not pectin, whilst only isolate IF17, a close relative to *S. alkaliphilus* DSM 42118^T^, hydrolyzed chitin. Four pine forest isolates degraded pectin, namely NA10a and SH15, SH56, and SH57, which are close relatives to *S. celluloflavus* NRRL B-2493^T^ *S. cocklensis* BK168^T^ and *S. halstedii* NBRC 12783^T^, respectively. In contrast, 12 of the 28 Atacama Desert isolates (43%) hydrolyzed pectin. Five of these strains showed activity indices which ranged from 12.6 to 21.4, namely isolates 1R2A7, 3HA10, and 1SCA21, which are phylogenetically close to *S. aquilus*, *S. galbus* and *S. tendae*, respectively, and isolates 2G9 and 4SCA5, members of putative novel *Streptomyces* species (Table 1). Similarly, isolate 1G2, a close relative of *S. flaveolus* NBRC 3715^T^, and isolate 2SCA1, a member of potentially novel *Kribbella* species were found to have activity indices of 9.4 and 8.3, respectively. The pine forest strain, isolate SH56, a close relative to *S. cocklensis* BK168^T^, had an activity index of 12.8.

Fifteen out of 28 (54%) isolates from the Atacama Desert soils and 10 out of 16 (63%) isolates from saline soil produced cellulases. The corresponding numbers from the northern and southern pine dune slopes were low at 25% and 23%, respectively. In general, the highest activity indices were shown by isolates from the desert and saline soils, as exemplified by eight Atacama Desert strains which had activity indices that ranged from 13.4 to 27.3 (Supplementary Table S3A). These isolates included 1R2A1 and 1G2, close relatives to *S. flaveolus* NBRC 3715^T^, 3HA10, and 2SCA1 mentioned earlier, and 5HA2, and 5SCA5 and 5SCA7, which are phylogenetically close to *S. mutabilis* NBRC 12800^T^ and *S. marokkonensis* Ap1^T^, respectively whilst 5R2A3 is closely related to *M. acroterricola* 5R2A7^T^.

Seven isolates from the saline soil had activity indices for cellulose degradation ranging from 9.4 to 17.1; three of them, IF11, IF15, and IF19, are close to *S. alkaliphius* DSM 42118^T^, two, OF4 and OF6 to *N. metallicus* KBS6^T^ whereas IT2 and IF7 were shown to be members, of prospective novel species of *Nocardiopsis* and *Streptomyces* (Table 1). Seven of the pine forest isolates showed activity indices for cellulase hydrolysis at or above 8.3, including NH28a and NL23, members, of prospective novel *Actinacidiphila* species; the remaining isolates, namely SL16, NL3, SH56, SH57, and NL35 are near neighbours of the type strains of *P. columellifera* subspecies *pallida*, *S. celluloflavus*, *S. cocklensis*, *S. halstedii*, and *S. yanii*, respectively. In contrast, isolates 1HA3, 2R2A4, and 3G5, all of which were assigned to the genus *Pseudonocardia*, did not degrade cellulose nor did they hydrolyze chitin, pectin or milk protein.

Twenty-six (23%) of the isolates from the sampling locations degraded chitin albeit with low activity indices (Supplementary Table S3). In marked contrast, all of the isolates from the saline soil hydrolyzed powdered milk, as did most of those from the Atacama Desert soils (57%); the corresponding results for isolates from the northern and southern pine dune slopes were 42% and 69%. High levels of proteolytic activity (activity indices at or above 12.0) were recorded for 11 of the isolates (9.6%), including 2G7 and NH7, close relatives of *K. flavida* DSM 17836^T^ and *S. celluloflavus* NRRL B-2493^T^, respectively, and isolate 2G9, a member of a prospective novel *Streptomyces* species. In turn, the eight strains from the pine forest sites showed pronounced proteolytic activity, namely isolates NF24, NF39 and NH9, NH15, NH16, SA16, and SL19 and SL24 which are close to the type strains of *K. herbaricolor*, *S. yanii*, *S. atratus*, *S. sanglieri*, and *P. columellifera* subspecies *pallida*, respectively. Similarly, isolates SF23, SF8, and SA8, which are close to the type strains of *P. columellifera*, *S. celluloflavus*, and *S. sanglieri,* respectively, were found to have activity indices ranging from 10.9 to 11.9. In addition, high indices of proteolytic activity, 11.1 and 11.6, were recorded for isolate IF15, a close relative to the type strain of *S. alkaliphilus*, and isolate 2SCA1, a member of a prospective novel *Kribbella* species.

Few of the isolates from the saline and Atacama Desert soils hydrolyzed urea. In contrast, 30 (83%) and 32 (91%) of those from the northern and southern pine dune slopes did so. Urease production in many of the pine forest strains was high, as exemplified by strains closely related to the type strains of *A. yanglinensis* (isolates NH21, SL5, SL10, SL22), *P. columellifera* subspecies *pallida* (isolates SA4, SF23, SL4, SL16, SL19, SL55, and SL24), *S. atratus* (isolates NH5 and NH16) and *S. celluloflavus* (isolates NA10a, SA7, SH11, SH15, and SL3).

### 3.7. Activity against fungal and oomycete plant pathogens

**Figure 3:**
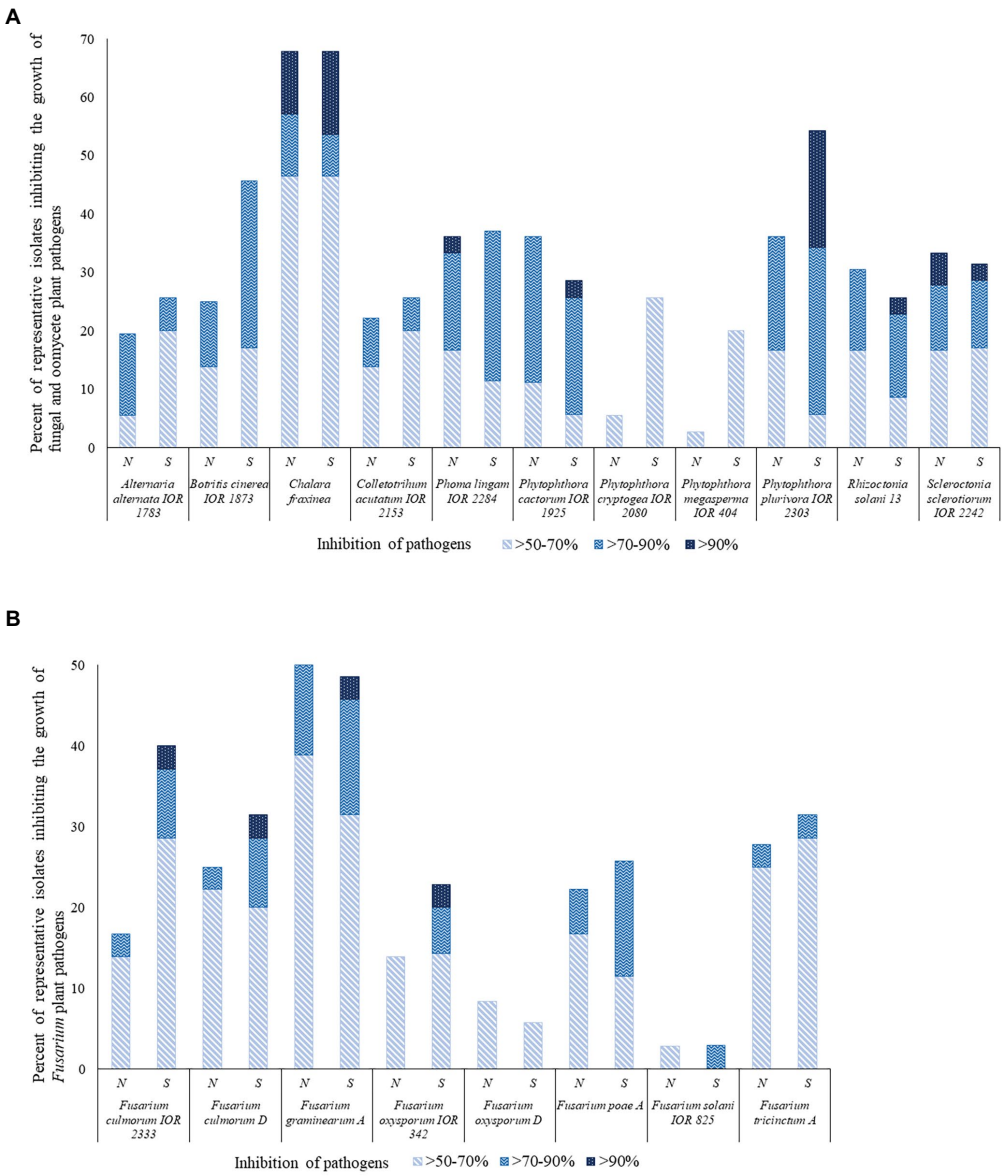
Representative isolates [%] inhibiting fungi and fungi-like organisms tested using a co-culture method. N and S, isolates from the northern and southern slopes of the inland dunes of the pine forest. **(A)** Fungal and fungal-like pathogens; **(B)** *Fusarium* species that are agents of a plant diseases.

Many of the representative isolates from the pine forest soils showed a remarkable ability to inhibit the growth of the fungal and oomycete plant pathogens, as shown in Figure 3 and Supplementary Table S4. In general, isolates from the southern slope of the inland pine dune showed more activity than those from the northern slope. Isolates from the southern inland dune were particularly active against *F. culmorum* IOR 2333*, F. culmorum* D*, F. graminearum* A, *F. oxysporum* IOR 342*, P, cactorum* IOR 1925, and *P. plurivora* IOR 2303. In contrast, a few isolates from the northern slope showed pronounced activity against *P. lignam* IOR 2284.

Sixty-two out of the 71 (87%) representative isolates from the pine forest sites showed inhibition indices over 50% against at least one of the 19 pathogens. The highest activity levels were recorded for *S. pinistramenti* SF28^T^ which inhibited the growth of 17 of the plant pathogens, including 11 where inhibition values fell within the range 82.4%–94.6% It is encouraging that results from this study are in good agreement with those reported by Świecimska et al. (2022). Isolate NA10a, which was found to have an identical 16S rRNA gene sequence similarity with *S. celluloflavus* NRRL B-2493^T^, inhibited the growth of 11 of the pathogens, as did isolate NH17, a member of a putative novel *Streptomyces* species. The growth of eight of the pathogens was markedly reduced by isolate SH15, a close relative of the type strain of *S. celluloflavus*; similar results were recorded for isolate NH28, another putatively novel *Streptomyces* strain.

Representatives of other genera shown to have an inhibition value >50% against some of the plant pathogens included isolates NH9 and NF39, close relatives of *K. herbaricolor* NBRC 12876^T^, and isolates NH22 and SF10, which share high or identical sequence similarities with *S. neutrinimicus* DSM 41755^T^ and *S. torunensis* NF37^T^, respectively. Isolates NH7 and SF9 can be added to this group, the former is a close relative to *S. celluloflavus* NRRL B-2493^T^, and the latter is a member of a putatively novel *Nocardia* species. Isolates showing high levels of activity against the plant pathogens were isolated from each of the horizons of the pine forest.

The *C. fraxinea*, *F. graminearum* A and *P. plurivora* IOR 2303 strains were particularly sensitive to some of the isolates, as shown in Supplementary Table S4. In contrast, few of the strains inhibited the growth of either *F. solani* IOR 825 or *F. oxysporum* D. The exceptions were *S. pinistramenti* SF28^T^ and isolate NA24, these strains inhibited the growth of the *F. solani* strain to varying degrees. Isolates NF24 and NF39, close associates to *K. herbaricolor* NBRC 12876^T^, inhibited the growth of *F. oxysporum* D, as did isolate NH17, one of the putatively novel *Streptomyces* species, and isolates SA20 and SH56, which were found to share high sequence similarities with *S. atratus* NNRL B-16927^T^ (99.79%) and *S. cocklensis* BK168^T^ (99.15%), respectively.

## 4. Discussion

### 4.1. Colour-group assignment, dereplication of isolates and phylogeny

The effectiveness of culture-dependent strategies designed to detect novel specialised metabolites of biotechnological interest tend to reflect the taxonomic diversity of filamentous actinomycetes isolated from extreme habitats (Goodfellow et al., 2018; Benaud et al., 2022; Pathom-aree et al., 2022). It is encouraging that in the present study filamentous actinomycetes chosen to represent colony types growing on selective isolation plates were recovered in a broad range of single-and multi-membered colour-groups given their ability to form distinctive pigments on oatmeal agar. Colour-groups have been used extensively as an index of actinomycete diversity in natural habitats, including extreme biomes (Goodfellow and Fiedler, 2010; Idris, 2016; Kusuma, 2020). It is interesting that in this study the most extensive actinomycete diversity was found in the litter and mineral horizons of the pine forest.

Confidence can be placed in the 16S rRNA gene sequence data, not least because few significant incongruities were found between whole-genome and 16S rRNA gene trees in an extensive genome-based classification of actinomycetes (Nouioui et al., 2018). The phylogenetic data showed that isolates representing each set of colour-groups had distinctive taxonomic profiles thereby underpinning the pioneering work of Williams and his colleagues who found that the distribution of actinomycete populations in different habitats was a product of different environmental variables, such as organic matter content, pH, temperature and water availability (Williams and Mayfield, 1971; Williams et al., 1972). Indeed, it is now known that in Atacama Desert soils intensive solar irradiation acts synergistically with desiccation in limiting the survival and growth of microbial life (Gómez-Silva, 2018).

Representative isolates from the high altitude, hyper-arid Atacama Desert soils and saline soil were assigned to the genera *Kribbella, Micromonospora, Modestobacter*, *Pseudonocardia,* and *Nocardiopsis*, respectively, results in good agreement with those from previous surveys (Idris, 2016; Meklat et al., 2020; Tsetseg, 2023). Similarly, acidotolerant and acidophilic actinomycetes from the pine forest sites assigned to the genera *Actinospica, Catenulispora, Kitasatospora, Nocardia* and *Streptacidiphilus* tallies with those from earlier studies (Golińska et al., 2023). Members of all of these taxa are known to synthesise bioactive compounds, including novel antibiotics (Takahashi, 2017; Hifnawy et al., 2020; Riahi et al., 2022; Golińska et al., 2023; Virués-Segovia et al., 2022).

The taxonomic status of individual colour-groups can be determined from the distribution of the reference strains known to represent specific genera or species (Atalan et al., 2000; Sembiring et al., 2000; Manfio et al., 2003). In this study, multi-membered colour-groups containing isolates from the Atacama Desert soils were found to belong to the genus *Streptomyces* and the four genera mentioned above. In turn, saline isolates assigned to multi-membered groups corresponded to or were closely related to *Nocardiopsis metallicus* (Schippers et al., 2002) and *Streptomyces alkaliterrae* (Świecimska et al., 2020) whilst ones forming single-membered colour-groups were most closely related to *Nocardiopsis halotolerans* (Al-Zarban et al., 2002), *Nocardiopsis flavescens* (Fang et al., 2011) and *Nocardipsis valiformis* (Yang et al., 2008). Similarly, multi-membered colour-groups encompassing isolates from the northern slope of the pine forest were equated with the genera *Actinacidiphila, Catenulispora, Kitasatospora, Pilimelia, Streptacidiphilus*, and *Streptomyces*. Corresponding multi-membered colour-groups composed of isolates from the southern slope of the pine forest were either equated with the genera *Actinacidiphila, Actinospica, Nocardia*, and *Streptomyces* or were close to or *bona fide* members of *Pilimelia columellifera* subspecies *pallida* (Vobis et al., 1986) and *Streptomyces celluloflavus* (Nishimura et al., 1953) Madhaiyan et al., 2020. These data provide further evidence that 16S rRNA gene sequencing remains a practical way of determining the taxonomic status of filamentous actinomycetes isolated from extreme habitats (Goodfellow et al., 2018; Singh and Dubey, 2018; Sharma and Thakur, 2020; Liu et al., 2021).

Over 40% of the isolates assigned to the colour-groups belonged to the genus *Streptomyces*. The highest number, 69%, were from the saline soil and the lowest, 27%, from the northern slope of the inland pine dune. These results are in line with those from previous studies where streptomycetes were shown to be the dominant component of extreme habitats (Bull and Asenjo, 2013; Jiang et al., 2018; Sharma and Thakur, 2020). In contrast, streptomycetes have not featured as major components of actinomycete communities in culture-independent studies, as exemplified by analyses of actinomycetes in Atacama Desert soils (Idris et al., 2017a; Bull et al., 2018) and diverse extreme biomes in Indonesia (Kusuma, 2020). These disparities can be attributed to biases in culture-independent methods, such as difficulties in extracting DNA from streptomycetes, choice of PCR primers, RNA copy numbers and PCR amplification (Kutchma et al., 1998; Klappenbach et al., 2000; Engelbrektson et al., 2010; Zielińska et al., 2017), to associated data handling issues (Claesson et al., 2010; Escobar-Zepeda et al., 2015), and to the use of isolation media that select for streptomycetes (Goodfellow and O’Donnell, 1989).

Several isolates assigned to the genus *Streptomyces* were found to belong to putatively novel species whereas others showed relatively low sequence similarities with the type strains of their closest phylogenetic neighbours or were close to streptomycetes that have rarely been isolated from natural habitats, such as *Streptomyces atratus* (Shibata et al., 1962), *Streptomyces cocklensis* (Kim et al., 2012), *Streptomyces mutabilis* (Preobrazhenskaya and Ryabova, 1957), *Streptomyces flaveolus* (Waksman, 1923; Waksman and Henrici, 1948) and *Streptomyces sanglieri* (Manfio et al., 2003). These results are particularly interesting as similar isolates from extreme habitats have been found to produce novel specialised metabolites (Donald et al., 2022), including *Streptomyces leeuwenhoeki* strains from hyper-arid Atacama Desert soils (Busarakam et al., 2014) which synthesise ansamycin and macrolactone polyketides (Goodfellow et al., 2018).

Twenty-two (19.1%) of representative isolates found to belong to putatively novel species were assigned to genera other than *Streptomyces*. Nearly half of them were most closely related to the type strains of *Streptomyces* species that were transferred to the genus *Actinacidiphila*, namely *A. alni, A. bryophytorum, A, paucisporea, A. rubida*, and *A. yanglinensis* (Madhaiyan et al., 2022) whereas others were most closely related to three validly named *Streptacidiphulus* species, *S. albus* (Kim et al., 2003), *S. carbonis* (Kim et al., 2003) and *S. hamsterleyensis* (Golińska et al., 2013). It seems likely that presumptively novel isolates shown to be most closely related to the type strains of *Streptomyces cocklensis* (Kim et al., 2012) and *Streptomyces ferralitis* (Saintpierre-Bonaccio et al., 2004) will be found to belong to the genus *Actinacidiphila* given the close phylogenetic relationships of these taxa with *Streptomyces* species recently transferred to this genus (Labeda et al., 2012, 2017). It is also interesting that isolate SA10 is most closely related to the type strain of *Actinospica* acidiphila (Cavaletti et al., 2006), an actinomycete found to merit generic status (Golińska et al., 2023). Similarly, presumptively novel isolates from the other sampling sites were found be most closely related to *Kribbella turkmenica* (Saygin et al., 2019), *Nocardiopsis flavescens* (Fang et al., 2011), *Nocardiopsis halotolerans* (Al-Zarban et al., 2002), and *Pseudonocardia xinjiangensis* (Xu et al., 1999; Huang et al., 2002). It is likely that some of the isolates found to share sequence similarities above the 99.0% threshold with the type strains of their immediate phylogenetic neighbours will be members of putatively novel species. Indeed, it has been shown that actinomycetes sharing very high and almost identical 16S rRNA gene sequence similarities can be classified into different species based on extensive polyphasic taxonomic studies, as shown in the case of closely related members of the genera *Gordonia*, *Micromonospora* and *Streptomyces* strains (Riesco et al., 2018, 2022; Kusuma et al., 2021).

The taxonomic data acquired in this study show that selective isolation, dereplication and initial characterization of representative isolates from diverse extreme biomes is a simple and practical way of selecting putatively novel and rare filamentous actinomycetes for exploitative biotechnology. In addition, these data underpin the rational of ecologically guided bioprospecting campaigns featuring actinomycetes (Mitra et al., 2011; Nalini and Prakash, 2017; Liu et al., 2021; Wang et al., 2022). Many of the isolates found to belong to rare and putatively novel taxa were from the litter and mineral horizons of the pine forest indicating that acidophilic and acidotolerant filamentous actinomycetes should feature more prominently in the search for novel bioactive compounds, especially given evidence that such strains are a source of novel antibiotics and acid-stable enzymes (Golińska et al., 2023). Further studies are also needed to determine whether litter and mineral horizons in coniferous woodlands contain characteristic actinomycete communities, as implied by Goodfellow and Dawson (1978).

### 4.2. Antimicrobial activity

There is a urgent need to find a new generation of antibiotics that are effective against multidrug-resistant microbial pathoges (Tacconelli et al., 2018), especially Gram-negative ones that cause high mortality rates in hospital acquired infections (Mehrad et al., 2015). It is encouraging that nearly 70% of the dereplicated isolates included in the antimicrobial screens showed activity against at least one of a panel of reference strains, as was the case in corresponding studies on isolates from extreme hyper-arid Atacama Desert soils where hit rates of 68% were recorded (Busarakam et al., 2014; Idris, 2016). These figures are much higher than those recorded in comparable studies on non-dereplicated isolates (Sengupta et al., 2015; Prieto-Davó et al., 2016; Priyadarshini et al., 2016). Further, the importance of growing dereplicated isolates on more than one production medium (Goodfellow and Fiedler, 2010) was underlined by instances where positive results were only reported for isolates grown on only one of the cultivation media.

The isolates from the pine forest sites, notably those from the southern inland pine dune, were especially effective in inhibiting the growth of the *E. coli* (34%), *K. pneumoniae* (28%), *P. aeruginosa* (10%), *P. mirabilis* (15%), and *S. infantis* (30%) strains following growth on one or both of the cultivation media. The most active isolates were *S. pinistramenti* SF28^T^ and those closely related to *P. columellifera* subspecies *pallida* and *S. celluloflavus*, as they inhibited the growth of all but one of the reference strains. In contrast, few, if any, of the isolates from the Atacama Desert and saline sites inhibited the growth of the *E. coli, K. pneumoniae*, and *P. mirabilis* strains. In contrast, the five *S. alkaliterrae* isolates from the saline soil inhibited the growth of the *P. aeruginosa* strain following growth on HA agar; isolate 1G2, a near relative of *S. flaveolus*, from the Atacama Desert soil also inhibited the growth of this reference strain when cultivated on ISP3 agar.

The ability of the isolates to inhibit the growth of the Gram-positive reference strains was evenly distributed across all of the sampling sites though none of the isolates from the saline soil were active against *S. aureus* PCM 2054 and only a putatively novel *Nocardiopsis* strain inhibited the growth of *B. subtilis* PCM 2021. Thirty-five of the isolates (30%) showed activity against the latter, the corresponding figures for the *M. luteus* and *S. aureus* reference strains were 53 (46%) and 38 (33%), respectively, following growth on at least one of the production media. Comparable results were recorded against the *B. subtilis* and *S. aureus* reference strains for dereplicated filamentous actinomycetes from high altitude Atacama Desert soils (Idris, 2016).

Most of the 25 isolates (22%) active against all of the Gram-positive reference strains were from the pine forest sites. These isolates included *S. pinistramenti* SF28^T^ and ones found to be closely related to *S. celluloflavus*, *P. columellifera* subspecies *pallida*, and *S. xanthochromogenes*; four of the remaining strains were assigned to putatively novel *Actinacidiphila* species and another two were members of prospective novel *Streptomyces* species. Similarly, two of the four corresponding isolates from the Atacama Desert soils were found to be putatively novel *Streptomyces* species; the remaining ones, isolates 3HA10 and 1SCA19, were most closely related to *Streptomyces galbus* (Frommer, 1959) and *Streptomyces purpurascens* (Lindenbein, 1952), respectively.

Most of the 33 isolates (29%) shown to be active against *C. albicans* ATCC 10231 were from the pine forest soils though the five *S. alkaliterrae* isolates from the saline soil also gave positive results. The 15 isolates from the pine forest which gave positive results following growth on ISP2 and ISP3 agar included five that were close to *S. celluloflavus*, two to *S. xanthochromogenes,* one to *P. columellifera* subspecies *pallida* (isolate NL28), and six belonging to presumptively novel *Actinacidiphila*, *Streptacidiphilus* and *Streptomyces* species. The final strain, isolate NH22, is phylogenetically close to the type strain of *Streptacidiphilus neutrinimicus*.

Little, if anything, is known about the antimicrobial activities of the taxa cited above though a strain from an acid mangrove soil identified as *S. celluloflavus* produced diverse specialised metabolites (Nguyan and Cao, 2022). Similarly, a soil isolate identified as *S. purpurascens* synthesised several bioactive compounds, notably rhodomycin C, which is particularly active against *B. subtilis* (Holkar et al., 2013). Further, a strain identified as *S. xanthochromogenes* showed antifungal activity (Singh et al., 2016), as did a *P. columellifera* subspecies *pallida* isolate which was active against fungi-causing superficial mycoses (Wypij et al., 2017).

### 4.3. Plant growth promoting features

Microbial inoculants are needed to promote plant growth and control plant diseases given challenges posed by climate change and sustainable agriculture (Debasis et al., 2019; Pacios-Michelena et al., 2021). Actinomycetes from extreme habitats are increasingly being seen as relevant in this respect given their role in nutrient recycling and promoting plant growth, as witnessed by their capacity to solubilise phosphate, synthesise phytohormones, notably IAA, lytic enzymes and siderophores (Boukhatem et al., 2022; Pathom-aree et al., 2023). The use of *Streptomyces venezuelae* as a biofertilizer, for example, increased maize production under drought conditions due to its ability to secrete high levels of IAA and L-aminocyclopropane (ACC; Chukwuneme et al., 2020); ACC is an immediate precursor of the gaseous hormone, ethylene. Plant growth promoting bacteria (PGPB) play an important role lowering plant stress by reducing ethylene levels by hydrolyzing ACC to ammonia, α-ketobutyrate and methionine thereby regulating ethylene production (Mulani et al., 2021); plants inoculated with ACC-deaminase producing bacteria are more resistant to abiotic and biotic stress (Gupta and Pandey, 2019). In the present study, representative isolates from the Atacama Desert and pine forest sampling sites produced ammonia and IAA, solubilised phosphate and synthesised siderophores. In contrast, none of those from the saline soil produced IAA or solubilised phosphate and only one, isolate OT1, a close relative to *Nocardiopsis valliformis*, formed ammonia. However, actinomycetes are known to promote the growth of plants adapted to saline conditions, as exemplified by an endophytic *Micromonospora chalcea* strain which enhanced the growth of *Salicornia bigelovii* (El-Tarabily et al., 2019).

In general, actinomycetes isolated from the pine forest were the most proficient in synthesising PGP-metabolites, as shown by five isolates that were most active in producing ammonia, namely *S. pinistramenti* SF28^T^, strains close to the type strains of *S celluloflavus* and *S. sanglieri* and ones considered to be members of presumptive novel species of *Streptacidiphilus* and *Streptomyces*. Isolates 1HA1 and 5R2A3, near relatives of *Kribbella italica* (Everest et al., 2015) and *Micromonospora acroterricola* (Carro et al., 2019), respectively, were particularly gifted in their ability to produce IAA, as was isolate SA8, another close relative of *S. sanglieri*.

Thirteen isolates were shown to be especially active in synthesising siderophores (activity indices 8.6–17.7). They included six from the Atacama Desert, namely ones phylogenetically close to *Streptomyces flaveolus*, *Streptomyces marokkonensis* (Bouizgarne et al., 2009), *Streptomyces mutabilis* (Preobrazhenskaya and Ryabova, 1957) and *Streptomyces tendae* (Ettlinger et al., 1958), the remaining strain, isolate 2G9, belongs to a putatively novel *Streptomyces* species most closely related to *Streptomyces paradoxus* (Krasil'nikov and Yuan, 1961; Goodfellow et al., 1986). The corresponding organisms from the pine forest sites were *C. pinisilvae* NF3, *S. pinistramenti* SF28^T^, isolates close to *S. celluloflavus* and *S. halstedii,* and isolate NH17, a presumptively novel *Streptomyces* species. The remaining isolates close to *S. celluloflavus* also produced siderophores, notably NA10a, SH15, SF8, and SL3, which showed activity indices ranging from 4.7 to 6.6. These results provide additional evidence that taxonomically diverse filamentous actinomycetes are a valuable source of iron-binding compounds (Franco-Correa and Chavarro-Anzola, 2016), many of which promote plant growth (Boukhatem et al., 2022).

### 4.4. Enzyme activity

Actinomycetes also promote plant growth by secreting hydrolytic enzymes, notably cellulases and chitinases, which convert insoluble polymers into nutrients which act as natural fertilizers (Jog et al., 2016; Nouioui et al., 2019). Novel hydrolytic enzymes are also a valuable resource for industrial processes (Mukhtar et al., 2017; Jin et al., 2019). Isolates from all of the sampling sites produced cellulases, chitinases, lipases and proteinases though pectinase activity was restricted mainly to ones from the Atacama Desert soils, notably isolates 1R2A7, 3HA10, and 1SCA21, which were found to be close relatives to the type strains *Streptomyces aquilus*, *S. galbus* and *S. tandae*, respectively, and 2G9 and 4SCA5, members of two prospective novel *Streptomyces* species. Several isolates showed a marked ability to produce cellulases (activity indices ≥15.0), mainly ones related to *M. acroterricola*, *N. metallicus, S. cocklensis*, *S. flaveolus, S. galbus*, *S. marokkonensis* and isolate IF7, which was assigned to a putatively novel *Streptomyces* species. Atacama Desert isolates also showed an ability to produce lipases (activity indices >10), as shown by strains closely related to *K. flavida*, *M. acroterricola*, *Pseudonocardia khuvsgulensis* (Ara et al., 2011), *Pseudonocardia rhizophila* (Li et al., 2010) and *S. mutabilis*; two additional strains, isolates 2SCA1 and 3G5, were shown to be putatively novel *Kribbella* and *Pseudonocardia* species, respectively.

The seventy-one isolates (62%) which secreted proteases included all of those from the saline soil. Isolates showing high or very high activity indices (>10) were recovered from all of the sampling sites. Most of the isolates were streptomycetes that either belonged or were closely related to *S. alkaliphilus*, *S. alkaliterrae*, *S. atratus*, *S. cellulofalvus*, *S. sanglieri*, *S. yanii*, and *S. xanthochromogenes* or, as in the case of isolate 2G9, belonged to a putatively novel *Streptomyces* species. The most highly active non-streptomycetes isolates were either closely related to the type strains of *K. herbaricolor* and *K. flavida* or represented a prospective novel *Kribbella* species. These results are in sync with those reported for many strains isolated from extreme habitats, as exemplified by a strain found to be closely related to the type strain of *Kribbella gitaiheensis* (Tsetseg and Badamgavar, 2021), a soil isolate (Guo L. et al., 2015).

It is not surprising that isolates from each of the sampling sites hydrolyzed urea, as this is common feature of streptomycetes. However, all of the most highly active strains were from the pine forest soils, including *S. pinistramenti* SF28^T^, and ones closely related to *S. atratus* and *S. yanii*. In addition, seven out of the nine isolates closely related to the type strain of *S. celluloflavus* gave strong reactions. Similarly, all but one of the isolates closely related to *P. columellifera* subspecies *pallida* gave highly positive results, the exception, isolate SF15, was negative. Most of the putatively novel *Actinacidiphila*, *Streptacidiphilus*, and *Streptomyces* isolates gave markedly positive results.

### 4.5. Activity against fungal and oomycete plant pathogens

New effective eco-friendly methods are needed to control phytopathogenic fungi, especially ones that reduce yields of staple crops (Law et al., 2017; Debasis et al., 2019; Boukhatem et al., 2022; Ebrahimi-Zarandi et al., 2022). Actinomycetes are to the fore amongst microbial control agents given their ability to produce antifungal compounds and siderophores, secrete enzymes that degrade fungal cell walls and compete for nutrients (Guo X. et al., 2015; Alblooshi et al., 2021). Taxonomically diverse actinomycetes, notably streptomycetes, inhibit the growth of phytopathogens, as exemplified by Abdelrahman et al. (2022) who found that streptomycetes from rhizosphere and soil samples in the Sudan inhibited the growth of the devastating oomycete pathogen *Phytophthora infestans*. Further, an actinomycete closely related to *Streptomyces spectabilis* inhibited the growth of 11 plant pathogens (Chen et al., 2018) whereas *Nocardiopsis* and *Streptomyces* were found to show *in vitro* and *in planta* activity against bacterial and fungal pathogens of carrots and tomatoes (Djebaili et al., 2021). In this context, it is remarkable that so many of the isolates from the litter and mineral horizons of the pine forest inhibited the growth of the fungal pathogens. Little is known about the functional roles of actinomycetes in acidic forest soils though it seems likely that they will compete with fungal populations for nutrients (Matthies et al., 1997; Rousk et al., 2009).

Taxonomically diverse isolates, albeit mainly streptomycetes, showed pronounced broad spectrum activity (>70% inhibition) against many of the fungal and oomycete pathogens, notably isolates showing high or identical sequence similarities with the type strains of *S. celluloflavus* and *S. xanthochromogenes*. The most active isolate in this respect was *S. pinistramenti* SF28^T^ which inhibited the growth of 17 of the 19 plant pathogens, a result that built upon the earlier work of Świecimska et al. (2022). These results provide further evidence, that streptomycetes from poorly studied extreme habitats have the ability to control the growth of plant pathogens (Suksaard et al., 2017; Pathom-aree et al., 2019; Qi et al., 2019).

Non-streptomycete isolates which showed either pronounced or notable broad spectrum activity against some of the fungal pathogens included ones closely related to *Catenulispora pinisilvae*, *Kitasatospora herbaricolor*, *Streptacidiphilus neutrinimicus* and *Streptacidiphilus torunensis,* and putatively novel isolates that were most closely related to the type strains of *Nocardia nova* and *Streptacidiphilus hamsterleyensis*. These results provide further evidence that isolates assigned to several actinomycete genera have the ability to inhibit the growth of fungal pathogens (Martínez-Hidalgo et al., 2015; Ebrahimi-Zarandi et al., 2022). Few of the isolates showed pronounced activity against the oomycetes, a notable exception was *S. pinistramenti* SF28^T^, which inhibited the growth of *P. cactorum* and *P. plurivora*; several isolates which shared high or identical sequence similarities with the type strains of *P. columellifera* subspecies *pallida* and *S. celluloflavus* also inhibited the latter.

## 5. Conclusion

Initial steps in natural product pipelines designed to discover new bioactive compounds from actinomycetes are often taken for granted given an understandable focus on outcomes, that is, the commercial exploitation of novel chemical compounds. Nevertheless, the selective isolation, characterization and screening of actinomycetes from unexplored or poorly studied extreme biomes highlights actinomycetes that can be prioritised in the search for new bioactive compounds using state-of-the-art technologies, such as genome mining, genetic engineering and procedures that allow rapid dereplication of chemical entities from complex biological extracts.

In the present study the importance of the early steps in the natural product pipeline was underpinned by the isolation of taxonomically diverse actinomycetes from three extreme ecosystems which produced a broad range of bioactive compounds. It was particularly significant that dereplicated isolates from the litter and mineral horizons of the pine forest included members of rare and novel genera which not only showed an extraordinary ability to inhibit the growth of diverse fungal and oomycete phytopathogens, but also inhibited the growth of members of Gram-negative taxa that are on the list of multidrug-resistant taxa highlighted by the World Health Organization. In addition, representatives of several genera, notably *Actinacidiphila*, *Pilimelia* and *Streptomyces,* not only inhibited the growth of a panel of microorganisms in the antimicrobial assays, but also produced compounds that promote plant growth. Another notable outcome of this study was the isolation of so many putatively novel species, especially ones belonging to the genera *Streptomyces* and the association of other isolates with rare validly named species belonging to poorly studied genera, such as *Actinacidiphila, Kribbella, Pilimelia* and *Streptacidiphilus*. Further work is now in the progress to build upon these developments.

Consequently, all-embracing culture-dependent studies such as the present one should be seen as an integral part of platforms designed to foster research collaboration on actinomycetes designed to address some of the critical challenges facing humankind, not least the need to find new antibiotics for multiple purposes and effective biofertilizers and bioinoculants for sustainable agricultural practices. It is also worth noting that enforceable measures are needed to safeguard actinomycete communities in fragile biomes given problems associated with habitat destruction and the effects of climate breakdown.

## Data availability statement

The raw data supporting the conclusions of this article will be made available by the authors, without undue reservation.

## Author contributions

PG and MG: conceptualization, writing and editing the manuscript. PG and MŚ: methodology and validation. MŚ: formal analysis, investigation, resources, data curation, writing—original draft preparation, visualisation, project administration, and funding acquisition. PG: supervision. All authors have read and agreed to the published version of the manuscript.

## Funding

This research was funded by Nicolaus Copernicus University, grant Nos. 90-SIDUB.6102.40.2021.G4NCUS1 and 1207-B and the APC was funded by IDUB NCU.

## Conflict of interest

The authors declare that the research was conducted in the absence of any commercial or financial relationships that could be construed as a potential conflict of interest.

## Publisher’s note

All claims expressed in this article are solely those of the authors and do not necessarily represent those of their affiliated organizations, or those of the publisher, the editors and the reviewers. Any product that may be evaluated in this article, or claim that may be made by its manufacturer, is not guaranteed or endorsed by the publisher.

## Footnotes

^1^http://eztaxon-e.ezbiocloud.net/
