# Supplementary material for: Generation of a high quality library of bioactive filamentous actinomycetes from extreme biomes using a culture-based bioprospecting strategy
Source: Front Microbiol. 2023 Jan 19;13:1054384. doi: 10.3389/fmicb.2022.1054384 (PMC9893292; doi:10.3389/fmicb.2022.1054384)
Supplement: Supplementary file 1 [file Data_Sheet_1.PDF]

Supplementary Material

Table S1. Assignment of isolates to colour-groups based on environmental samples from the four sampling locations following growth on oatmeal agar (ISP3) at 28°C for 4 weeks. The numbers given in parenthesis denotes colour codes from the ISCC-NBS charts (Kelly, 1958). Codes in bold indicate representatives of colour-groups included in the 16S rRNA gene sequencing, antimicrobial and enzyme screening studies.

Colour groups	Aerial spore mass/aerial hyphal pigments	Substrate mycelial pigments	Colour of diffusible pigments	Number of isolates	Isolate codes
**(A) Isolates from the hyper-arid Atacama Desert soils**					
1	Medium gray (265)	Moderate yellow green (106)	None	7	5G1, 5HA5, 5R2A4, 5R2A6, 5SCA3, **5SCA5, 5SCA7**
2	Absent	Strong orange yellow (68)	None	7	1SCA3, 1SCA8, 3HA4, 3R2A2, **5R2A3, 5R2A7** ^{T}, 5R2A8
3	White (263)	Pale greenish yellow (104)	None	6	**1HA3**, 2SCA3, 3G3, **3G5**, 3HA16, **4R2A1**
4	Absent	Black (267)	None	6	**1G4**^{T}, **1G6**^{T}, **1G14**, **1G50**, **1G51**, **1G52**
5	White (263)	Brilliant greenish yellow (98)	None	5	**1HA1**, **2G7**, **2SCA1**, 4G1, 4G6,
6	Dark gray (266)	Dark greenish yellow (103)	None	5	1G3, 1R2A9, 1SCA15, 1SCA17, **1SCA21**
7	Dark gray (266)	Moderate yellow green (120)	None	5	**1G2**, 2G12, **3G6**, **5SCA4**, 5SCA8
8	Greenish gray (155)	Moderate greenish yellow (102)	None	4	3G7, 3HA9, **3HA10**, 3HA13
9	Strong bluish green (160)	Brilliant yellow green (116)	None	4	1G16, **1SCA19**, 1SCA20, 2G17
10	Medium gray (265)	Deep greenish yellow (100)	None	3	**2G9**, 2R2A1, **5HA2**
11	Medium gray (265)	Moderate olive (107)	None	3	1G8, **1R2A1**, 3HA19
12	Medium gray (265)	Strong greenish yellow (99)	None	3	**1R2A7**, 2G6, **4SCA5**
13	Medium gray (265)	Moderate greenish yellow (102)	None	1	**2R2A4**
**(B) Isolates from the saline soil adjacent to Lake Lonar**					
1	Absent	Moderate yellow (87)	Pale purple (227)	5	**OF1**^{T}, **OF2**, **OF3**, **OF7**, **OF8**
2	Light gray (264)	Moderate olive (107)	None	3	**IF11**, **IF17**, **IF19**
3	Yellowish white (92)	Moderate yellowish brown (77)	Light yellowish brown (76)	2	**IF7, OF5**
4	White (263)	Strong yellow (84)	Grayish yellow (90)	2	**OF4**, **OF6**
5	White (263) scant	Yellowish white (92)	None	1	**IF12**

6	Absent	Yellowish gray (93)	None	1	**IT2**
7	Pale orange yellow (73)	Moderate olive brown (95)	Dark grayish yellow (91)	1	**IF15**
8	White (263)	Strong orange yellow (68)	Light orange yellow (70)	1	**OT1**
**(C) Isolates from the litter and mineral horizons of the northern slope of the inland dune of the pine forest**					
1	Absent	Yellowish white (92)	None	9	NA2, NA12, NA14, NA25, NA28, **NF10**, NF37a, NH26, NL25
2	Pale green (149)	Dark greenish yellowish green (151)	Grayish yellowish green (122)	9	NF5, NF15, **NF24**, NF35, NF36, NF37, NF40, **NH9**, NH18
3	Strong purplish red (255)	Greenish white (153)	None	9	**NF3**, NF13, **NF23**, NF32, **NH11**^{T}, NH26a, **NL8**^{T}, **NL13**, NL37
4	Pale yellowish green (121)	Dark grayish yellow (91)	Dark yellow (88)	8	**NA24,** NL3a, NL5, NL14, **NL21**, NL30*, NL34, NL38
5	Light greenish gray (154)	Dark grayish brown (62)	Grayish olive (110)	4	**NH15**, **NH5**, **NH16**, **NL35**
6	Greenish white (153)	Grayish yellow (90)	None	3	**NA4**, **NA21**, **NL15**
7	Light bluish gray (190)	Dark bluish gray (192)	Light grayish olive (109)	2	**NF27**, **NL23**
8	Greenish white (153)	Light olive gray (112)	Grayish greenish yellow (105)	2	**NH21**, **NH27**
9	Greenish white (153)	Light olive gray (112)	Dark grayish yellow (91)	2	NL27, **NL28**
10	Greenish white (153)	Grayish olive (110)	Light grayish olive (109)	2	**NF39**, NL20
11	Pinkish gray (10)	Grayish reddish brown (46)	Dark red (16)	2	**NH28a**, **NL16**
12	Greenish white (153)	Grayish greenish yellow (105)	None	2	**NF20**, NH4
13	Light greenish gray (154)	Light greenish gray (154)	Light olive (106)	2	**NA10a**, **NH17**
14	Greenish white (153)	Yellowish gray (93)	Grayish yellow (90)	2	NA27, **NH14**
15	Greenish white (153)	Light brown gray (63)	Grayish greenish yellow (105)	1	**NA13**
16	Bluish white (189)	Dark bluish gray (192)	Light grayish olive (109)	1	**NA19a**
17	Yellowish white (92)	Grayish yellow (90)	Grayish yellow (90)	1	**NF22**
18	White (263)	Grayish yellow (90)	Strong greenish yellow (99)	1	**NH7**
19	White (263)	Grayish yellow (90)	Moderate greenish yellow (102)	1	**NH28**
20	Yellowish white (92)	Moderate yellow (87)	Moderate yellow (87)	1	**NH22**
21	Greenish white (153)	Light yellowish brown (76)	None	1	**NL3**
**(D) Isolates from the litter and mineral horizons of the southern slope of the inland dune of the pine forest**					
1	Moderate yellow green (120)	Brilliant greenish yellow (98)	Brilliant yellow green (116)	24	SA1, SA3, SA5, **SA7**, SA18, SF1, SF2, **SF8**, SF18, SF20, SF21, SF25, SH3, SH6,

					SH9, **SH11**, SH12, SH13, SH23, SH25, SH26, SH28, SH55, **SL3**
2	White (263)	Vivid yellow (82)	None	12	**SA4,** SF12, **SF15, SH24, SL4**, SL6, SL9, SL15, SL20, **SL24** SL26*, **SL55**
3	Moderate yellowish green (136)	Deep yellow green (118)	None	8	**SA8,** SA13, SA14, **SA16**, SA25, SA26, SA27, SA28
4	Dark yellowish green (137)	Moderate olive green (125)	None	7	SA9, SA11, SA12, SA17, SA19, SA21, **SF28^{T}**
5	White (263)	Dark greenish yellow (103)	Brilliant yellow green (116)	7	**SF23**, SF30, SL1, SL8, **SL16, SL19**, SL27
6	White (263)	Strong yellow green (117)	None	4	**SA10**, SA24, SH16, SH17,
7	Moderate yellowish green (136)	Strong greenish yellow (99)	Brilliant yellowish green (130)	4	**SA23**, SA30, **SF17,** SF29
8	Light pink (4)	Deep orange (51)	Strong orange (50)	3	**SL5, SL10, SL22**
9	White (263)	Very light yellowish green (134)	None	3	**SF9, SF13, SH20**
10	Moderate green (145)	Dark grayish olive green (128)	Grayish yellow (90)	3	**SL7**, SL29, **SL52**
11	Moderate yellowish green (136)	Light olive brown (94)	None	3	SL12, **SL13**, SL21
12	White (263)	Brilliant yellow green (116)	None	2	SL28, **SL54**
13	White (263)	Vivid greenish yellow (99)	None	1	**SF4**
14	White (263)	Moderate greenish yellow (102)	None	1	**SH57**
15	Moderate yellowish green (136)	Moderate yellow green (120)	None	1	**SA20**
16	Moderate yellowish green (136)	Strong yellow green (117)	None	1	**SF10**
17	White (263)	Moderate yellowish green (136)	None	1	**SH15**
18	Pale pink (7)	Deep orange (51)	Strong orange (50)	1	**SH56**

*Isolates that produced melanin pigments. ^{T}, type strain.

Table S2. Classification of representative isolates assigned to colour-groups based on the distribution of reference strains included in the 16S rRNA gene sequence analysis. Isolates in bold are shown with their closest phylogenetic neighbours. Those with sequence similarities at or below the threshold of 99.0% can be considered to be members of putatively novel species.

**(A) Colour-groups composed of isolates from the hyper-arid Atacama Desert soils**	
1	**5SCA5** and **5SCA7** (*Streptomyces marokkonensis* Ap1^{T}, both 99.50%), 5G1, 5HA5, 5R2A4, 5R2A6 and 5SCA3
2	***Micromonospora acroterricola* 5R2A7^{T}** and **5R2A3** (*M. acroterricola* 5R2A7^{T}, 99.72%), 1SCA3, 1SCA8, 3HA4, 3R2A2 and 5R2A8
3	**1HA3** (*Pseudonocardia khuvsgulensis* MN08-A0297^{T}, 99.36%), **3G5** *Pseudonocardia xinjiangensis* AS 4.1538^{T}, 97.81%), **4R2A1** (*Pseudonocardia rhizophila* YIM 67013^{T}, 99.64%), 2SCA3, 3G3 and 3HA16
4	***Modestobacter altitudinis* 1G4^{T}**, **1G51** and **1G52** (*M. altitudinis* 1G4^{T}, both 100%), ***Modestobacter excelsi* 1G6^{T}**, **1G14** and **1G50** (*M. excelsi* 1G6^{T}, 100 and 99.86% respectively)
5	**2G7** (*Kribbella flavida* DSM 17836^{T}, 99.43%), **1HA1** (*Kribbella italica* BC637^{T}, 99.36%), **2SCA1** (*Kribbella turkmenica* 16K104^{T}, 98.94%), 4G1 and 4G6
6	**1SCA21** (*Streptomyces tendae* ATCC 19812^{T}, 99.15%), 1G3, 1R2A9, 1SCA15, 1SCA17
7	**5SCA4** (*Streptomyces albogriseolus* NRRL B-1305^{T}, 99.43%), **3G6 (***Streptomyces bungoensis* DSM 41781^{T}, 99.51%), **1G2 (***Streptomyces flaveolus* NBRC 3715^{T}, 99.72%), 2G12 and 5SCA8
8	**3HA10** (*Streptomyces galbus* DSM 40089^{T}, 99.37%), 3G7, 3HA9 and 3HA13
9	**1SCA19** (*Streptomyces purpurascens* NBRC 13077^{T}, 99.15%), 1G16, 1SCA20 and 2G17
10	**5HA2** (*Streptomyces mutabilis* NBRC 12800^{T}, 99.79%), **2G9** (*Streptomyces paradoxus* NBRC 14887^{T}, 98.79%) and 2R2A1
11	**1R2A1** (*Streptomyces flaveolus* NBRC 3715^{T}, 99.72%), 1G8 and 3HA19
12	**4SCA5** (*Streptomyces camponoticapitis* 2H-TWYE14^{T}, 97.58%), **1R2A7** (*Streptomyces aquilus* GGCR-6^{T}, 99.28%) and 2G6
13	**2R2A4** (*Pseudonocardia rhizophila* YIM 67013^{T}, 99.72%)
**(B) Colour-groups composed of isolates from the saline soil adjacent to Lake Lonar**	
1	***Streptomyces alkaliterrae* OF1^{T}**, **OF2**, **OF3**, **OF7** and **OF8** (*S. alkaliterrae* OF1^{T}, all 100%)
2	**IF11**, **IF17**, **IF19** (*Streptomyces alkaliphilus* DSM 42118^{T}, 99.42, 99.52, 99.50%, respectively)
3	**IF7** (*Streptomyces cahuitamycinicus* 13K301^{T}, 97.24%) and **OF5** (*S. alkaliterrae* OF1^{T}, 99.79%)
4	**OF4** and **OF6** (*Nocardiopsis metallicus* KBS6^{T}, 100 and 99.86%, respectively)
5	**IF12** (*Nocardiopsis halotolerans* DSM 44410^{T}, 98.72%)
6	**IT2** (*Nocardiopsis flavescens* CGMCC 4.5723^{T}, 97.45%)
7	**IF15** (*S. alkaliphilus* DSM 42118^{T}, 99.36%)
8	**OT1** (*Nocardiopsis valliformis* DSM 45023^{T}, 99.50%)
**(C) Colour-groups composed of isolates from the litter and mineral horizons of the northern slope of the inland dune of the pine forest**	
1	**NF10** (*Streptacidiphilus torunensis* NF37^{T}, 100%), NA2, NA12, NA14, NA25, NA28, NF37a, NH26 and NL25
2	**NF24** and **NH9** (*Kitasatospora herbaricolor* NBRC 12876^{T}, 99.71, 99.43%, respectively), NF5, NF15, NF35, NF36, NF37, NF40 and NH18
3	***Catenulispora pinisilvae* NH11^{T}**, **NF3** and **NL13** (*C. pinisilvae* NH11^{T}, 100 and 99.79%, respectively), ***Catenulispora pinistramenti* NL8^{T}** and **NF23** (*C. pinistramenti* NL8^{T} 99.86%), NF13, NF32, NH26a and NL37
4	**NA24** and **NL21** (*Streptomyces xanthochromogenes* NRRL B-5410^{T}, 100 and 99.93%, respectively), NL3a, NL5, NL14, NL30, NL34, NL38
5	**NH5** and **NH16** (*Streptomyces atratus* NRRL B-16927^{T}, 100 and 99.79%, respectively), **NH15** and **NL35** (*Streptomyces yanii* NBRC 14669^{T}, 99.20 and 99.12%, respectively)

6	**NA4** (*Streptacidiphilus albus* NBRC 100918^{T}, 97.73%), **NL15** (*Streptacidiphilus carbonis* DSM 41754^{T}, 98.44%) and **NA21** (*Streptacidiphilus hamsterleyensis* HSCA 14^{T}, 92.01%)
7	**NL23** (*Actinacidiphila paucisporea* CGMCC 4.2025^{T}, 98.62%) and **NF27** (*Actinacidiphila yanglinensis* 1307^{T}, 98.14%)
8	**NH21** and **NH27** (*Actinacidiphila yanglinensis* 1307^{T}, 99.07 and 99.22%, respectively)
9	**NL28** (*Pilimelia columellifera* subsp. *pallida* MB-SK 8^{T}, 99.64%) and NL27
10	**NF39** (*Kitasatospora herbaricolor* NBRC 12876^{T}, 99.64%) and NL20
11	**NH28a** (*Actinacidiphila alni* D65^{T}, 98.50%) and **NL16** (*Actinacidiphila bryophytorum* NEAU-HZ10^{T}, 98.48%)
12	**NF20** (*Streptacidiphilus torunensis* NF37^{T}, 99.86%) and NH4
13	**NA10a** (*Streptomyces celluloflavus* NRRL B-2493^{T}, 100%) and **NH17** (*Streptomyces luteireticuli* NBRC 13422^{T}, 92.32%)
14	**NH14** (*Streptacidiphilus torunensis* NF37^{T}, 100%) and NA27
15	**NA13** (*Streptacidiphilus durhamensis* FSCA67^{T}, 99.64%)
16	**NA19a** (*Streptacidiphilus durhamensis* FSCA67^{T}, 99.86%)
17	**NF22** (*Streptacidiphilus hamsterleyensis* HSCA 14^{T}, 99.56%)
18	**NH7** (*Streptomyces celluloflavus* NRRL B-2493^{T}, 99.86%)
19	**NH28** (*Streptomyces paludis* GSSD-12^{T}, 98.13%)
20	**NH22** (*Streptacidiphilus neutrinimicus* DSM 41755^{T}, 99.78%)
21	**NL3** (*Streptomyces celluloflavus* NRRL B-2493^{T}, 100%)
**(D) Colour-groups composed of isolates from the litter and mineral horizons of the northern slope of the inland dune of the pine forest**	
1	**SF8**, **SH11** and **SL3** (*Streptomyces celluloflavus* NRRL B-2493^{T}, all 100%), **SA7** (*Streptomyces celluloflavus* NRRL B-2493^{T}, 99.79%), SA1, SA3, SA5, SA18, SF1, SF2, SF18, SF20, SF21, SF25, SH3, SH6, SH9, SH12, SH13, SH23, SH25, SH26, SH28 and SH55
2	**SA4**, **SF15**, **SH24**, **SL4** and **SL24** (*Pilimelia columellifera* subsp. *pallida* MB-SK 8^{T}, all 99.71 or 99.72%), **SL55** (*Pilimelia columellifera* subsp. *pallida* MB-SK 8^{T}, 99.0%), SF12, SL6, SL9, SL15, SL20 and SL26
3	**SA8** and **SA16** (*Streptomyces sanglieri* NBRC 100784^{T}, 100 and 99.79%), SA13, SA14, SA25, SA26, SA27 and SA28
4	***Streptomyces pinistramenti* SF28^{T}**, SA9, SA11, SA12, SA17, SA19 and SA21
5	**SF23**, **SL16** and **SL19** (*Pilimelia columellifera* subsp. *pallida* MB-SK 8^{T}, all 99.71%), SF30, SL1, SL8 and SL27
6	**SA10** (*Actinospica acidiphila* GE134766^{T}, 98.07%), SA24, SH16 and SH17
7	**SA23** and **SF17** (*Actinacidiphila yanglinensis* 1307^{T}, 99.36 and 99.50%, respectively), SA30 and SF29
8	**SL5**, **SL10** and **SL22** (*Actinacidiphila yanglinensis* 1307^{T}, 98.70, 98.84 and 98.78%, respectively)
9	**SF9** (*Nocardia nova* NBRC 15556^{T}, 98.85%), **SF13** and **SH20** (*Nocardia vinacea* NBRC 16497^{T}, 98.22 and 98.26%)
10	**SL7** and **SL52** (*Actinacidiphila bryophytorum* NEAU-HZ10^{T}, 98.33 and 98.61%) and SL29
11	**SL13** (*Actinacidiphila rubida* 13C15^{T}, 97.49%), SL12 and SL21
12	**SL54** (*Streptomyces ferralitis* SFOp68^{T}, 97.65%) and SL28
13	**SF4** (*Streptomyces celluloflavus* NRRL B-2493^{T}, 100%)
14	**SH57** (*Streptomyces halstedii* NBRC 12783^{T}, 99.79%)
15	**SA20** (*Streptomyces atratus* NRRL B-16927^{T}, 99.79%)
16	**SF10** (*Streptacidiphilus torunensis* NF37^{T}, 100%)
17	**SH15** (*Streptomyces celluloflavus* NRRL B-2493^{T}, 99.79%)
18	**SH56** (*Streptomyces cocklensis* BK168^{T}, 99.15%)

^{T}, type strain.

Table S3. Colour-groups composed of isolates from the sampling sites which produce hydrolytic enzymes and compounds that support plant growth. Codes in bold are isolates that show 16S rRNA sequence similarities with their nearest neighbours at or below the 99.0% sequence similarity threshold.

Isolates	Compounds promoting plant growth					Production of					
	Production of			Siderophore activity*	Phosphate solubilization*						
	Ammonia	HCN	IAA			Cellulases	Chitinases	Lipases	Pectinases	Proteases	Ureases
**(A) Representative isolates from the hyper-arid Atacama Desert soils**											
1G2	-	-	+	13.2	0.0	24.1	0.0	4.9	9.4	9.7	-
1G4^T	-	-	-	0.0	0.0	0.0	0.0	0.0	0.0	0.0	-
1G6^T	++	-	+	0.0	0.0	0.0	0.0	0.0	0.0	0.0	-
1G14	-	-	-	0.0	0.0	0.0	0.0	0.0	0.0	0.0	-
1G50	-	-	-	0.0	0.0	0.0	0.0	0.0	0.0	0.0	-
1G51	-	-	-	0.0	0.0	0.0	0.0	0.0	0.0	0.0	-
1G52	-	-	-	0.0	0.0	0.0	0.0	0.0	0.0	0.0	-
1HA1	-	-	+++	3.1	0.0	1.1	0.0	6.0	0.0	8.2	-
1HA3	-	-	-	2.1	0.0	0.0	0.0	16.0	0.0	0.0	-
1R2A1	++	-	-	17.7	0.0	18.7	0.6	6.3	5.6	9.7	+
1R2A7	++	-	-	0.0	0.5	4.6	0.0	7.9	21.4	5.8	++
1SCA19	-	-	+	0.0	0.0	4.6	0.0	1.8	0.0	5.0	-
1SCA21	+	-	++	15.9	0.7	9.0	0.0	3.6	12.6	8.0	++
2G7	-	-	-	0.6	0.0	1.4	0.0	12.5	0.0	12.5	-
**2G9**	**-**	-	**-**	**9.3**	**0.0**	**3.6**	**0.0**	**3.2**	**17.3**	**12.0**	**+**
2R2A4	-	-	-	1.3	0.0	0.0	0.0	19.6	0.0	0.0	-
**2SCA1**	**-**	-	+	**4.6**	**0.0**	**13.4**	**0.6**	**10.7**	**8.3**	**11.6**	**+**
**3G5**	**-**	-	**-**	**0.6**	**0.0**	**0.0**	**0.0**	**13.4**	**0.0**	**0.0**	**-**
3G6	-	-	-	0.3	0.0	0.0	0.0	0.0	0.0	0.0	-
3HA10	+	-	+	0.0	0.0	27.3	0.0	2.2	18.7	5.8	-
4R2A1	-	-	-	0.3	0.0	0.0	0.0	10.0	0.0	0.0	-

**4SCA5**	-	-	+	**0.0**	**0.0**	**0.0**	**0.8**	**3.1**	**19.0**	**8.7**	-
5HA2	-	-	-	14.4	0.0	17.5	0.0	19.4	4.6	4.5	+
5R2A3	+	-	+++	0.0	0.1	18.0	0.0	10.7	0.0	10.7	-
5R2A7^{T}	++	-	++	0.0	0.0	0.0	0.0	0.0	0.0	0.0	-
5SCA4	-	-	-	7.4	0.0	5.8	1.1	7.0	4.0	7.0	-
5SCA5	-	-	+	0.0	0.0	15.0	0.8	4.3	2.7	4.3	+
5SCA7	-	-	-	11.5	0.0	16.5	0.8	5.1	3.0	5.1	+
**(B) Representative isolates from the saline soil adjacent to Lake Lonar**											
**IF7**	-	-	-	**0.3**	**0.0**	**17.0**	**0.0**	**1.4**	0.0	**9.8**	-
IF11	-	-	-	0.0	0.0	9.5	0.0	3.0	0.0	7.1	-
**IF12**	-	-	-	**0.0**	**0.0**	**6.8**	**0.0**	**0.9**	0.0	**3.1**	-
IF15	-	-	-	0.0	0.0	13.9	0.0	2.0	0.0	11.1	-
IF17	-	-	-	1.9	0.0	6.3	0.7	1.8	0.0	5.0	-
IF19	-	-	-	0.0	0.0	12.4	0.0	2.0	0.0	7.1	-
**IT2**	-	-	-	**0.0**	**0.0**	**9.4**	**0.0**	**0.4**	0.0	**1.4**	-
OF1^{T}	-	-	-	0.0	0.0	0.0	0.0	2.7	0.0	2.7	++
OF2	-	-	-	0.0	0.0	0.0	0.0	3.6	0.0	6.3	+
OF3	-	-	-	0.0	0.0	0.0	0.0	3.6	0.0	10.1	+
OF4	-	-	-	0.0	0.0	17.1	0.0	1.8	0.0	5.7	-
OF5	-	-	-	2.5	0.0	0.0	0.0	2.9	0.0	4.9	-
OF6	-	-	-	0.0	0.0	15.6	0.0	2.4	0.0	4.6	-
OF7	-	-	-	0.0	0.0	0.0	0.0	2.7	0.0	3.6	+
OF8	-	-	-	0.0	0.0	0.0	0.0	2.3	0.0	6.3	-
OT1	+	-	-	0.0	0.0	5.4	0.0	1.9	0.0	3.1	-
**(C) Representative isolates from the litter and mineral horizons of the northern slope of the inland dune of the pine forest**											
**NA4**	-	-	-	**0.0**	**0.0**	**0.0**	**0.0**	**4.5**	**0.0**	**0.0**	**+++**
NA10a	+++	-	-	5.7	0.0	0.0	0.0	3.6	1.1	3.4	+++
NA13	+	-	-	0.0	0.0	0.0	0.0	0.0	0.0	0.0	+++
NA19a	++	-	-	0.0	0.0	0.0	0.0	0.0	0.0	0.0	++

**NA21**	**-**	**-**	**-**	**0.0**	**0.0**	**0.0**	**0.0**	**0.0**	**0.0**	**0.0**	**+++**
NA24	++	-	+	0.0	0.0	0.0	0.0	0.0	0.0	4.9	+++
NF3	-	-	+	9.6	0.0	0.0	0.7	10.9	0.0	0.0	+
NF10	-	-	++	0.3	0.0	0.0	0.0	0.0	0.0	0.0	-
NF20	+	-	++	0.3	0.0	0.0	0.0	0.0	0.0	0.0	-
NF22	-	-	+	0.3	0.0	0.0	0.0	0.5	0.0	0.0	+
NF23	-	-	++	0.0	0.0	0.0	0.0	0.9	0.0	0.0	++
NF24	+	-	-	4.1	1.4	0.0	0.0	0.0	0.0	17.1	+
**NF27**	**-**	**-**	**++**	**0.0**	**0.0**	**2.2**	**0.0**	**1.0**	**0.0**	**0.0**	**-**
NF39	-	-	-	3.7	1.4	0.0	0.0	3.8	0.0	13.4	-
NH5	+	-	-	0.0	0.0	5.8	1.3	4.5	0.0	9.9	+++
NH7	-	-	-	8.6	0.0	0.0	1.5	3.6	0.0	12.0	+++
NH9	++	-	-	2.8	0.6	0.0	0.0	2.1	0.0	13.0	++
NH11^{T}	-	-	++	1.1	0.0	0.0	0.0	1.0	0.0	0.0	+
NH14	+	-	+	0.0	0.0	0.0	0.0	0.9	0.0	0.0	+
NH15	-	-	-	0.0	0.0	2.9	1.7	4.5	0.0	20.6	+++
NH16	-	-	-	3.3	0.0	2.1	1.4	3.2	0.0	17.8	+++
**NH17**	**+**	**-**	**-**	**8.6**	**0.0**	**0.0**	**0.7**	**4.0**	**0.0**	**6.7**	**+++**
NH21	-	-	-	0.0	0.0	0.0	0.0	0.0	0.0	0.0	+++
NH22	-	-	-	0.0	0.0	0.0	0.0	0.5	0.0	0.0	++
NH27	-	-	-	0.0	0.0	0.0	0.0	1.0	0.0	0.0	-
**NH28**	**+++**	**-**	**-**	**0.8**	**0.0**	**0.0**	**1.3**	**3.6**	**0.0**	**5.4**	**+++**
**NH28a**	**-**	**-**	**-**	**0.3**	**0.0**	**9.0**	**1.3**	**3.6**	**0.0**	**4.4**	**+++**
NL3	++	-	+	0.3	0.0	50.0	0.0	4.7	0.0	0.0	-
NL8^{T}	-	+	++	0.0	0.0	0.0	0.0	0.9	0.0	0.0	+
NL13	-	-	++	0.0	0.0	0.0	0.0	0.0	0.0	0.0	++
**NL15**	**+++**	**-**	**+**	**0.3**	**0.0**	**0.0**	**0.0**	**1.8**	**0.0**	**0.0**	**++**
**NL16**	**+**	**-**	**++**	**0.0**	**0.0**	**0.0**	**0.0**	**0.0**	**0.0**	**0.0**	**++**
NL21	++	-	++	0.0	1.1	3.5	0.0	8.3	0.0	10.3	++
**NL23**	**-**	**-**	**-**	**0.0**	**0.0**	**8.3**	**0.0**	**0.0**	**0.0**	**0.0**	**+**

NL28	-	-	-	0.0	0.0	0.0	0.4	3.4	0.0	2.4	++
NL35	++	-	-	0.7	0.0	15.4	1.3	3.3	0.0	10.5	+++
**D) Representative isolates from the litter and mineral horizons of the southern slope of the inland dune of the pine forest**											
SA4	-	-	+	0.0	0.0	0.0	0.0	4.0	0.0	3.2	+++
SA7	++	-	-	8.8	0.0	0.0	0.0	2.8	0.0	0.9	+++
SA8	-	-	+++	0.0	0.0	0.0	1.0	3.1	0.0	10.9	+++
**SA10**	**-**	**-**	**-**	**0.3**	**0.0**	**2.0**	**0.0**	**8.2**	**0.0**	**0.0**	**+++**
SA16	+++	-	-	6.7	0.0	0.0	0.8	3.2	0.0	13.7	+++
SA20	-	-	-	0.0	0.0	3.6	3.1	4.0	0.0	6.5	+++
SA23	+	-	+	1.4	0.0	7.4	0.0	0.5	0.0	2.7	+++
SF4	-	-	-	1.3	0.0	0.0	0.0	7.4	0.0	3.2	+
SF8	+	-	-	6.6	1.6	0.0	0.0	3.0	0.0	10.9	+++
**SF9**	**-**	**-**	**-**	**0.0**	**0.0**	**0.0**	**0.0**	**4.5**	**0.0**	**0.0**	**++**
SF10	+	-	-	0.3	0.0	0.0	0.0	0.0	0.0	0.0	+
**SF13**	**+**	**-**	**-**	**0.3**	**0.0**	**0.0**	**0.0**	**0.0**	**0.0**	**0.0**	**-**
SF15	+	-	+	3.5	0.0	0.0	0.0	5.3	0.0	2.7	-
SF17	+	-	++	0.0	0.0	2.7	0.0	1.8	0.0	0.0	+
SF23	+	-	-	1.2	0.0	0.0	0.6	2.7	0.0	11.9	+++
SF28^T	+++	-	-	11.3	0.0	0.0	0.9	4.5	0.0	10.8	+++
SH11	+	-	-	9.6	0.0	0.0	0.0	2.9	0.0	9.9	+++
SH15	+	-	-	4.7	1.1	0.0	0.0	0.0	0.6	9.6	+++
**SH20**	**+**	**-**	**-**	**0.0**	**0.0**	**0.0**	**0.0**	**3.6**	**0.0**	**0.0**	**+++**
SH24	-	-	-	6.8	0.0	0.0	0.0	4.1	0.0	4.1	+++
SH56	-	-	+	0.6	0.0	25.2	0.0	0.5	12.8	4.7	+
SH57	-	-	-	14.2	0.0	9.9	1.0	1.9	2.1	8.0	-
SL3	+	-	-	5.8	1.5	0.0	1.0	2.5	0.0	7.7	+++
SL4	+	-	+	0.0	0.0	0.0	0.0	6.7	0.0	1.9	+++
**SL5**	**-**	**-**	**-**	**0.3**	**0.0**	**2.0**	**0.0**	**0.0**	**0.0**	**1.8**	**+++**
**SL7**	**+**	**-**	**-**	**0.3**	**0.0**	**0.0**	**0.0**	**0.4**	**0.0**	**0.0**	**+++**

**SL10**	-	-	-	**0.3**	**0.0**	**0.0**	**0.0**	**4.0**	**0.0**	**0.0**	+++
**SL13**	+	-	-	**0.0**	**0.0**	**0.0**	**0.0**	**0.4**	**0.0**	**0.0**	+++
SL16	-	-	-	0.0	0.0	9.0	0.0	0.0	0.0	8.1	+++
SL19	+	-	+	0.0	0.0	0.0	0.8	3.1	0.0	12.7	+++
**SL22**	-	-	-	**0.0**	**0.0**	**0.0**	**0.0**	**0.0**	**0.0**	**0.0**	+++
SL24	+	-	-	0.0	0.0	0.0	0.8	2.2	0.0	13.9	+++
**SL52**	+	-	-	**0.3**	**0.0**	**0.0**	**0.0**	**0.0**	**0.0**	**4.5**	+++
**SL54**	-	-	+	**0.3**	**0.0**	**0.0**	**0.0**	**0.0**	**0.0**	**0.0**	+++
**SL55**	+	-	-	**3.8**	**0.0**	**0.0**	**0.0**	**3.8**	**0.0**	**7.5**	+++

Key: -, no production; +, low production; ++, moderate production; +++, strong production.
*Hydrolysis, siderophore and phosphate solubilization values are given as activity indices that were calculated as follows: Wact = Sh^2 (Sc × t) where Sh indicates the diameter of the hydrolysis zones, Sc the colony diameters and t the time of incubation (Hrynkiewicz et al., 2010).
Wact <1, very low activity; 1-5, low activity; >5-10, good activity; >10-20, high activity; >20, very high activity.

Atacama Desert isolates giving negative results: 1G4^T, 1G14, 1G50, 1G51, 1G52. ^{T}, type strain.

Table S4. Inhibition of fungal and oomycete plant pathogens by selected isolates from the pine forest sites based on a co-culture method. Bold print denotes isolates which showed 16S rRNA sequence similarity values below the 99.0% threshold for delineating actinomycete species.

**Pathogens**	**Extent of inhibition shown by isolates**		
	**>50-70%**	**>70-90%**	**>90%**
**Representative isolates from the litter and mineral horizons of the northern slope of the inland dune of the pine forest**			
**(A) Fungi**			
Alternaria alternata IOR 1783	NA24, NF20,	NA10a, NH7, **NH17**, **NH28**	None
Botrytis cinerea IOR 1873	NF24, NH9, **NH17**, **NL16**, NL21	NA10a, NA24, NH7, **NH28**	None
Chalara fraxinea	**NA4**, NA13, **NA21**, NF24, NH5, NH9, NH15, NH16, NH22, **NH28a**, **NL15**, NL28, NL35	NA24, NF39, **NH28**	NA10a, NH7, **NH17**
Colletotrichum acutatum IOR 2153	NF20, NF24, NF39, NH7, NH9	NA10a, **NH17**, **NH28**	None
Fusarium culmorum IOR 2333	NA10a, NF24, NF39, NH7, NL21	**NH17**	None
Fusarium culmorum D	NA10a, NA24, NF20, NF24, NF39, NH7, **NH28**, NL21	**NH17**	None
Fusarium graminearum A	NA13, **NA21**, NF3, NF24, **NF27**, NF39, NH9, NH11^{T}, NH15, NH27, **NH28**, NL13, **NL15**, NL21	NA10a, NA24, NH7, **NH17**	None
Fusarium oxysporum IOR 342	NA10a, NH7, **NH17**, **NH28**, NL28	None	None
Fusarium oxysporum D	NF24, NF39, **NH17**	None	None
Fusarium poae A	NA10a, NA24, NF24, NF39, **NH28**, NL21	NH7, **NH17**	None
Fusarium solani IOR 825	NA24	None	None
Fusarium tricinctum A	NA24, NF20, NF39, NH7, **NH17**, NH22, **NH28**, **NL16**, NL21	NA10a	None
Phoma lingam IOR 2284	NA13, NA24, NF22, NH5, NH15, NL35	NA10a, NF39, NH7, NH9, NH14, **NH17**, **NH28**	NF24
Rhizoctonia solani 13	NA13, NF24, NH5, NH9, NH15, NH16	NA10a, NF39, NH7, **NH17**, **NH28**	None
Sclerotinia sclerotiorum IOR 2242	NH7, NH14, NH15, NH27, **NH28**, **NL16**	NA10a, NA24, **NH28a**, NL21	**NA21**, NF3

**(B)** ***Oomycetes***			
Phytophthora cactorum IOR 1925	NF20, **NF27**, NH15, NL35	NA10a, NA24, NF24, NF39, NH7, NH9, **NH17**, **NH28**, NL21	None
Phytophthora cryptogea IOR 2080	NA10a, **NH28**	None	None
Phytophthora megasperma IOR 404	**NH28**	None	None
Phytophthora plurivora IOR 2303	NA24, NF24, NH9, NH21, NH22, NL21	NA10a, NA19a, NF39, NH7, NH16, **NH17**, **NH28**	None
**Representative isolates from the litter and mineral horizons of the southern slope of the inland dune of the pine forest**			
**(A) Fungi**			
Alternaria alternata IOR 1783	SA7, **SF9**, SF10, SH11, SH15, SL3, **SL13**	SF8, SF28^{T}	None
Botrytis cinerea IOR 1873	SF23, SH24, SH56, SH57, SL3, **SL10**	SA7, SA16, SF8, **SF9**, SF10, SF28^{T}, SH11, SH15, **SL13**, **SL54**	None
Chalara fraxinea	SA4, SA16, SA20, SF4, SF10, SF15, SF17, SF23, SF28^{T}, SL4, SL19, SL24, **SL55**	SH11, SH56	SA7, SF8, SH15, SL3
Colletotrichum acutatum IOR 2153	SA7, SF8, SH11, SH15, SF10, **SL13**, **SL52**	**SF9**, SL3	None
Fusarium culmorum IOR 2333	SA20, SA23, SF4, SF8, SF15, SF17, SH11, SH24, SH56, SH57	SA7, SH15, SL3	SF28^{T}
Fusarium culmorum D	SA8, SA20, SF17, SH11, SH15, SL3, **SL54**	SA7, SF8, SH56	SF28^{T}
Fusarium graminearum A	SA8, SA20, SA23, SF4, SF15, SF17, SH11, SL4, **SL13**, **SL54**, **SL55**	SA7, SF8, SH15, SH56, SL3	SF28^{T}
Fusarium oxysporum IOR 342	SA7, SF8, **SF9**, SH15, SL3	SF23, SH57	SF28^{T}
Fusarium oxysporum D	SA20, SH56	None	None
Fusarium poae A	SA20, SF10, SF28^{T}, SH56	SA7, SF8, SH11, SH15, SL3	None
Fusarium solani IOR 825	None	SF28^{T}	None
Fusarium tricinctum A	SA7, SA8, SA20, SF8, SF17, SF28^{T}, SH11, SH15, SL3, **SL54**	SH56	None

Phoma lingam IOR 2284	SA16, SA20, SA23, **SL54**	SA7, SF8, **SF9**, SF10, SF28^{T}, SH11, SH15, SL3, **SL13**	None
Rhizoctonia solani 13	SA20, SH11, **SL54**	SA7, SF8, SH15, SH56, SL3	SF28^{T}
Sclerotinia sclerotiorum IOR 2242	SA7, SA16, SF8, **SF9**, SH56, SL3	SA8, SH11, **SL10**, **SL54**	SF28^{T}
**(B) Oomycetes**			
Phytophthora cactorum IOR 1925	SA4, SH15	SA7, **SA10**, SF8, **SF9**, SF10, SF17, SL3, **SL13**	SF28^{T}
Phytophthora cryptogea IOR 2080	SA7, SF8, SF10, SF28^{T}, SH11, SH15, SH56, SL3, **SL13**	None	None
Phytophthora megasperma IOR 404	SA7, SF8, SF10, SF28^{T}, SH11, SH15, SL3	None	None
Phytophthora plurivora IOR 2303	SA23, SH57	SA8, SA20, SF4, SF8, SF17, SH15, SH56, SL3, **SL5**, **SL22**	SA4, SA7, SF15, SF28^{T}, SH11, SH24, SL4

*The % inhibition of the growth of the fungi and oomycetes was calculated using the following formula: I (%) = (C-T/C) × 100, where C is the diameter of pathogen growth in the control sample and T the diameter of pathogen growth in each of the co-cultures.

Antifungal and oomycete activities of *C. pinisilvae* NH11^{T} and NF3, and *S. pinistramenti* SF28^{T} were reported previously by Świecimska et al. (2021a and 2022). Activity against *Chalara fraxinea* was not tested for strains NF3, **NF27**, NH11^{T}, NL3, NL13, **NL16**, NL21, **NL23** (from the northern slope of the pine forest) or SH57, **SL5**, **SL10**, **SL13**, **SL22**, **SL52**, **SL54** (from the southern slope of the pine forest) as they not grown on the medium used for cultivation of this pathogen. ^{T}, type strain.
